# Higher plane of nutrition pre-weaning enhances Holstein calf mammary gland development through alterations in the parenchyma and fat pad transcriptome

**DOI:** 10.1186/s12864-018-5303-8

**Published:** 2018-12-11

**Authors:** M. Vailati-Riboni, R. E. Bucktrout, S. Zhan, A. Geiger, J. C. McCann, R. M. Akers, J. J. Loor

**Affiliations:** 1 Department of Animal Sciences and Division of Nutritional Sciences, University of Illinois, Urbana, 61801 USA; 2 College of Animal Science and Technology, Sichuan Agricultural University, Chengdu, Sichuan People’s Republic of China; 3 Department of Dairy Science, Virginia Tech, Blacksburg, VA 24061 USA

**Keywords:** Mammary gland, Milk replacer, Transcriptome, Calf

## Abstract

**Background:**

To reduce costs of rearing replacement heifers, researchers have focused on decreasing age at breeding and first calving. To increase returns upon initiation of lactation the focus has been on increasing mammary development prior to onset of first lactation. Enhanced plane of nutrition pre-weaning may benefit the entire replacement heifer operation by promoting mammary gland development and greater future production.

**Methods:**

Twelve Holstein heifer calves (< 1 week old) were reared on 1 of 2 dietary treatments (*n* = 6/group) for 8 weeks: a control group fed a restricted milk replacer at 0.45 kg/d (R, 20% crude protein, 20% fat), or an accelerated group fed an enhanced milk replacer at 1.13 kg/d (EH, 28% crude protein, 25% fat). At weaning (8 weeks), calves were euthanized and sub-samples of mammary parenchyma (PAR) and mammary fat pad (MFP) were harvested upon removal from the body. Total RNA from both tissues was extracted and sequenced using the Illumina HiSeq2500 platform. The Dynamic Impact Approach (DIA) and Ingenuity Pathway Analysis (IPA) were used for pathway analysis and functions, gene networks, and cross-talk analyses of the two tissues.

**Results:**

When comparing EH vs R 1561 genes (895 upregulated, 666 downregulated) and 970 genes (506 upregulated, 464 downregulated) were differentially expressed in PAR and MFP, respectively. DIA and IPA results highlight a greater proliferation and differentiation activity in both PAR and MFP, supported by an increased metabolic activity. When calves were fed EH, the PAR displayed transcriptional signs of greater overall organ development, with higher ductal growth and branching, together with a supportive blood vessel and nerve network. These activities were mediated by intracellular cascades, such as AKT, SHH, MAPK, and Wnt, probably activated by hormones, growth factors, and endogenous molecules. The analysis also revealed strong communication between MFP and PAR.

**Conclusion:**

The transcriptomics and bioinformatics approach highlighted key mechanisms that mediate the mammary gland response to a higher plane of nutrition in the pre-weaning period.

**Electronic supplementary material:**

The online version of this article (10.1186/s12864-018-5303-8) contains supplementary material, which is available to authorized users.

## Background

Replacement heifer rearing accounts for approximately 20% of annual farm costs [1–3]. To reduce costs, research has focused on: 1) lowering age at breeding and first calving, and 2) enhancing mammary development before first lactation to increase returns during the productive life of the cow. The first strategy appears the easiest and fastest to implement, being that puberty is tightly associated with body weight (**BW**), and Holstein heifer pubertal BW is relatively constant (250–280 kg or 40–50% of mature weight [4]). In fact, Tozer and Heinrichs [5] estimated a reduction of 4.3% of heifer rearing costs by decreasing age at first calving by 1 month, mainly due to a shortening of the non-productive portion of a heifer’s life by encouraging earlier herd entry and productivity.

To reach puberty weight earlier, producers must increase feed allowance in the pre-pubertal period. However, when this was done immediately after weaning, mammary parenchymal (**PAR**) tissue mass and DNA content in Holstein calves decreased by 23 and 32%, respectively [6]. The detrimental effects of increased pre-pubertal nutrient intake (starting post-weaning) on mammary development have long been recognized [7–10], and recently [6] confirmed in various studies [11–16]. Furthermore, the negative effect on mammary development may translate into poorer first lactation performance, as a recent study confirmed that heifers fed at a greater rate produced 14% less milk compared with controls [17].

It is well-accepted that events occurring pre-weaning can have lasting effects throughout a dairy cow’s life [18]. Recent data have indicated that the negative correlation between gain and mammary development may be the opposite in this period to what is observed in the pre-pubertal, post-weaning period [19–22]. Thus, an enhanced pre-weaning plane of nutrition (targeting greater gains) increases PAR and mammary fat pad (**MFP**) weight, DNA content of the mammary PAR, and total mammary PAR DNA [23]. Furthermore, increasing pre-weaning average daily gain by 1 kg/d was associated with an increase of 1000 kg or more in milk yield [24].

The mechanisms by which an increase in plane of nutrition pre-weaning enhances mammary development and consequent first lactation performance remain unclear. Therefore, the objective of this study was to obtain a general and holistic view through high-throughput mRNA sequencing of the mammary MFP and PAR transcriptome in Holstein heifer calves fed two distinct planes of nutrition in the pre-weaning period. By identifying genes altered by the dietary treatment, we could uncover molecular pathways and determine possible mediators of the mammary gland response to pre-weaning plane of nutrition. We hypothesized that an enhanced pre-weaning plane of nutrition positively alters mammary gland transcription by upregulating pathways involved in tissue growth and development. Furthermore, we hypothesized that a higher plane of nutrition could promote the inter-tissue communication between PAR and MFP. The effects of an improved plane of nutrition on general body growth and the development of multiple organs, mammary gland included, are reported elsewhere [23, 25].

## Methods

### Animal handling and experiment design

This experiment was conducted under the review and approval of the Virginia Polytechnic Institute and State University (**VT**) Institutional Animal Care and Use Committee (#14–045-DASC). The experimental design and animal handling were previously described [25]. Briefly, twelve Holstein heifer calves (6.0 ± 2 d old, 39.0 ± 4.4 kg at the time of arrival, and ≥ 5.5 mg/dL of total serum protein) were purchased from a single commercial producer (located ~ 90 miles from campus), brought to the VT Dairy Farm between May and June of 2014), and randomly assigned to two treatments. The control group was fed 0.45 kg/calf of milk replacer (**MR**) per day containing 20% crude protein (**CP**) and 20% fat (a restricted MR, **R**). The accelerated group was fed 1.13 kg of MR per day at 28% CP and 25% fat (an enhanced MR, **EH**). Starter feed was introduced at the end of week 4 and kept similar between treatments. Milk replacer was reduced in both treatments to 50% at week 8, to induce weaning. All calves were housed individually with ad libitum access to water. At weaning, calves were euthanized and their whole mammary glands were removed, weighed and dissected. A summary of phenotypic responses obtained and already published [25] is presented in Table 1.

**Table 1:** Summary of Holstein heifer calf (*n* = 6/treatment) performance when fed during the pre-weaning period (week 1 to 8 of life) an enhanced (EH) (1.08 kg of powder/calf/day, 28.9% crude protein, 26.2% fat, DM basis) or restricted (R; represents the industry standard or control) milk replacer (0.44 kg of powder/head/day, 20.9% crude protein, 19.8% fat, DM basis)

Parameters	R^1^	EH^2^	%^3^
*Milk replacer*			
DMI^4^ (kg/d)	0.44^a^	1.02^b^	131.82
CP^5^ intake (kg/d)	0.09^a^	0.30^b^	233.33
Fat intake (kg/d)	0.09^a^	0.27^b^	200.00
*Starter feed*			
DMI (g/d)	286.00^a^	237.00^b^	−17.13
VCP intake (g/d)	73.30^a^	60.90^b^	−16.92
Fat intake (g/d)	11.50^a^	9.50^b^	−17.39
NDF^6^ intake (g/d)	56.60^a^	46.90^b^	−17.14
ADF^7^ intake (g/d)	26.40^a^	21.90^b^	−17.05
*Growth*			
BW^8^ week 0 (kg)	39.80	39.40	−1.01
BW week 8 (kg)	51.29^a^	73.71^b^	43.70
ADG^9^ week 1 (kg/d)	−0.06^a^	0.36^b^	700.00
ADG week 7 (kg/d)	0.41^a^	1.00^b^	143.90
ADG week 8 (kg/d)	0.35^a^	0.31^b^	−11.43
ADG tot (kg/d)	0.20^a^	0.60^b^	208.21
Feed:Gain	1.33^a^	1.42^b^	6.77
Hip Height week 8 (cm)	87.90^a^	94.30^b^	7.28
*Slaughter*			
Carcass (kg)	48.60^a^	77.60^b^	59.67
Whole mammary gland (g)	66.10^a^	255.20^b^	286.08
Whole mammary gland (g/kg of BW)	1.34^a^	3.32^b^	147.76
Trimmed mammary gland (g)	37.80^a^	197.60^b^	422.75
Trimmed mammary gland (g/kg BW)	0.39^a^	1.30^b^	233.33
Mammary parenchyma (g)	1.42^a^	10.46^b^	636.62
Mammary parenchyma (g/kg BW)	0.02^a^	0.07^b^	250.00
Mammary fat pad (g)	29.20^a^	172.80^b^	491.78
Mammary fat pad (g/kg BW)	0.30^a^	1.13^b^	276.67
*Mammary fat pad*			
Total protein (g)	0.46^a^	2.11^b^	358.70
Protein (mg/g)	15.60^a^	12.20^b^	−21.79
Total DNA (mg)	4.10^a^	22.50^b^	448.78
DNA (mg/g)	0.14	0.13	−7.14
Total fat (g)	3.27^a^	116.00^b^	3447.40
Fat (mg/g)	112.00^a^	667.00^b^	495.54
*Mammary parenchyma*			
Total protein (g)	0.20^a^	1.36^b^	580.00
Protein (mg/g)	14.10	13.00	−7.80
Total DNA (mg)	2.70^a^	20.40^b^	655.56
DNA (mg/g)	1.87	1.95	4.28
Total fat (g)	0.15	2.10	1300.00
Fat (mg/g)	106.00	201.00	89.62

^1^R = restricted plane of nutrition (0.44 kg of powder/calf per day, 20.9% crude protein, 19.8% fat, DM basis)

^2^EH = enhanced plane of nutrition (1.08 kg of powder/head per day, 28.9% crude protein, 26.2% fat, DM basis)

^3^% = percentage increase or decreased in EH compared to R

^4^*DMI* dry matter intake

^5^*CP* crude protein

^6^*NDF* neutral detergent fiber

^7^*ADF* acid detergent fiber

^8^*BW* body weight

^9^*ADG* average daily gain

### Sample collection and slaughter procedures

A detailed description of sample collection can be found elsewhere [23, 25]. Calves at weaning (8 weeks) were euthanized at VT’s Veterinary Facility (approximately 1 mile from their housing) using a commercial phenobarbital solution administered intravenously (Fatal-Plus, 10 mg/kg of BW, Vortech Pharmaceuticals, Dearborn, MI), and subsequently exsanguinated. Pieces of PAR and MFP (~ 13.0 mg) were sampled from the mammary gland upon removal from the body, frozen by immersion in liquid nitrogen, and stored at − 80 °C.

### RNA extraction, library construction, and sequencing

Tissue was weighted (PAR, ~ 0.10 g; MFP, ~ 0.20 g), immediately placed in QIAzol Lysis Reagent (cat#79306, Qiagen) (1.20 mL) and homogenized using a Mini-Beadbeater-24 (cat#112011, Biospec Products Inc.) with two 30 s cycles, and 1 min incubation on ice in between the cycles. Samples were then centrifuged for 10 min at 12,000×g and 4 °C, and the supernatant was transferred to a separate tube and mixed with Chloroform (cat#C298, Fisher Chemical) (0.24 mL). After centrifugation for 15 min at 12,000×g and 4 °C, the aqueous phase was transferred to a new tube, mixed with 100% Ethanol (cat#2701, Decon Labs; 0.90 mL), and total RNA was cleaned using miRNeasy mini kit columns (cat# 217004, Qiagen) following manufacturer’s protocols. During purification, genomic DNA was removed using the RNase-Free DNase Set (cat#79254, Qiagen). Quantity and purity were determined using a NanoDrop ND-1000 (NanoDrop Technologies Inc.), while integrity was assessed via a Fragment Analyzer™ (Advance Analytical). All samples had an RQN score greater than 8.0. RNA samples were stored at − 80 °C until analysis.

RNA-Seq cDNA libraries were constructed using total RNA isolated from both MFP and PAR sampled at weaning (week 8) slaughter. The Illumina TruSeq Stranded mRNA Sample Prep kit was used for single-end read library construction following the manufacturer’s instructions with mRNA enrichment. Complete details of this procedure are available in Additional file 1. Libraries were pooled together and multiplexed across 4 flow cell lanes in the Illumina HiSeq4000 (Illumina Inc., San Diego, CA) platform to obtain an average of 20–30 million reads per sample.

### Transcriptome sequencing data processing and statistical analysis

Single-end reads were first filtered using Trimmomatic 0.33 [26] with a minimum quality score of 28 (i.e., base call accuracy of 99.84%) leading and trailing with a minimum length of 30 bp long and subsequently checked using FastQC 0.11.4 (Babraham Institute, Cambridge, UK). Reads were then mapped to the *Bos taurus* UMD 3.1.1 reference genome (1/29/16 NCBI release) using default settings of STAR 2.5.1b [27] with the quantMode option for gene counts. Further data analysis was conducted using R. 3.2.4 (R Core Team, 2016). Reads uniquely assigned to a gene were used for subsequent analysis. After accounting for high expression genes and library size differences using trimmed mean of M-values normalization in edgeR [28], genes were filtered if 4 samples did not have > 1 count per million mapped reads. Normalization of reads was conducted using the voom variance stabilization function in limma [29]. Differential expression analysis was conducted in limma using a single factor model which included the main effect of diet (2 levels). Raw *P*-values were adjusted using the false discovery rate (**FDR**) method [30]. Differences in transcript profiles (differentially expressed genes, **DEG**) were considered significant at an FDR-adjusted *P* < 0.05. The focus of this manuscript is on the overall differences in mammary gland transcriptome in response to the main effect of diet; that is, EH vs R.

### Bioinformatics analyses

#### Kyoto encyclopedia of genes and genomes (KEGG) pathways analysis

The dynamic impact approach (**DIA**) was used for KEGG pathway analysis of DEG. The detailed methodology of DIA is described elsewhere [31]. Briefly, the whole DEG data set with Entrez gene ID, FDR (< 0.05), fold-change (**FC**), and *P*-value (< 0.05) was uploaded to DIA. For the analyses, pathways with a minimum of 30% annotated genes in the transcriptome dataset versus the whole genome, and at least 4 genes were selected. Furthermore, pathways related to KEGG category “Human diseases” and Organismal system subcategories “Digestive system”, “Excretory system”, and “Sensory system” were not considered as pertinent to the analyzed tissues.

#### Function analysis and transcription regulator discovery

Ingenuity pathway analysis (**IPA**) software (https://www.qiagenbioinformatics.com/products/ingenuity-pathway-analysis/) was used to identify major affected functions and analyze the upstream regulators and their connections with other downstream genes that were differentially expressed. To identify only highly significant functions and upstream regulators, a list of DEG with FC > |1.5| and *P*-values (< 0.05) was uploaded to IPA, and a core analysis was run using default settings. Functions with *p*-value < 0.05 and z-score > |2.0| were considered significant, and upstream regulators were considered significant with overlap-*P* value < 0.05 and z-score > |2.0|.

#### Tissue interaction and crosstalk

The cross-talk between MFP and PAR was performed using the network capability of IPA as previously described [32]. To highlight how the dietary treatment affects such interactions, DEG (in the EHvsR comparison) considered to code for secreted proteins encompassed the cytokine and growth factor categories, while DEG coding for proteins considered to be receptors that might be able to “sense” the secreted proteins were those in G-protein coupled receptor, ligand-dependent nuclear receptor, transcription regulator, and transmembrane receptor categories. Networks between tissues were built using the IPA Knowledge base. The knowledge base was restricted to tissue and cell line categories “Mammary gland”, “Breast cancer cell lines”, and “Other cell line” when analyzing the effect of MFP secreted molecules on PAR, or to the categories “Adipose”, “Adipocytes”, and “Other cell line” when analyzing the effect of PAR secreted molecules on MFP. Furthermore, when analyzing the involvement of infiltrated immune cells in the tissue crosstalk, the categories were limited to “Immune cells”, “Immune cell lines”, and “Other cell lines”, regardless of the direction of the crosstalk.

### Real-time qPCR and statistical analysis

The results from RNA-seq were validated via qPCR for a selected panel of genes representing the top most upregulated (*n* = 4) or downregulated (n = 4) genes within each tissue. Complete details of primer design, qPCR analysis, and performance are available in Additional file 1. Briefly, the qPCR performed was SYBR Green-based, and results were calculated via the 2^-ΔΔCt^ method. *MTG1*, *PPP1R11*, and *RPS15A* were used as internal controls. Results were subjected to ANOVA and analyzed using repeated measures ANOVA with PROC MIXED within SAS (v9.4). To validate sequencing results, data from each tissue were independently analyzed, and the statistical model included diet as fixed effect. The Kenward-Roger (KR) statement was used for computing the denominator degrees of freedom. Data were considered significant at a *P* ≤ 0.05 using the PDIFF statement in SAS. The comparison with the RNA sequencing results is reported in Table 2.

**Table 2:** mRNA expression of target genes selected for validation of sequencing results. Genes were among the top up and downregulated genes in mammary parenchyma or fat pad of Holstein heifer calves (*n* = 6/treatment) fed pre-weaning (week 1 to 8 of life) an enhanced (EH) (1.08 kg of powder/head/day, 28.9% crude protein, 26.2% fat, DM basis) or restricted (R; represents the industry standard or control) milk replacer (0.44 kg of powder/head/day, 20.9% crude protein, 19.8% fat, DM basis)

Target	qPCR – 2^-ΔΔCt^		RNA-seq		
	FC^a^	*P*-value	FC^a^	FDR^b^	*P*-value
*Mammary parenchyma*					
*ABCC11*	−11.65	0.0259	−3.67	0.047	0.00410
*ADAM12*	2.56	0.0072	3.40	0.026	0.00010
*ASPHD1*	4.87	0.0473	3.60	0.046	0.00402
*LOC100335879*	4.95	0.0075	3.74	0.046	0.00395
*MPZ*	−9.71	0.0202	−3.80	0.029	0.00045
*PLN*	−17.14	0.0319	−3.93	0.037	0.00173
*SCN7A*	−13.56	0.0186	−3.37	0.038	0.00203
*SEZ6L*	8.14	0.0256	4.26	0.038	0.00195
*Mammary fat pad*					
*CAV3*	−2.64	0.0019	− 2.20	0.012	0.00009
*ELN*	6.26	0.0001	2.50	0.01	0.00003
*FCGR2A*	8.71	0.0007	2.70	0.007	0.00001
*LOC781726*	7.46	0.0970	3.72	0.019	0.00044
*PLIN4*	6.37	<.0001	2.46	0.012	0.00012
*PLPPR4*	−3.95	0.0345	−2.38	0.035	0.00165
*RPRML*	−2.16	0.0042	−2.51	0.042	0.00237
*WDR66*	−6.97	0.0015	−2.60	0.012	0.00009

^a^FC = fold change of EH vs R

^b^FDR = false discovery rate

## Results

### RNA sequencing and gene expression analyses

**Fig. 1:**
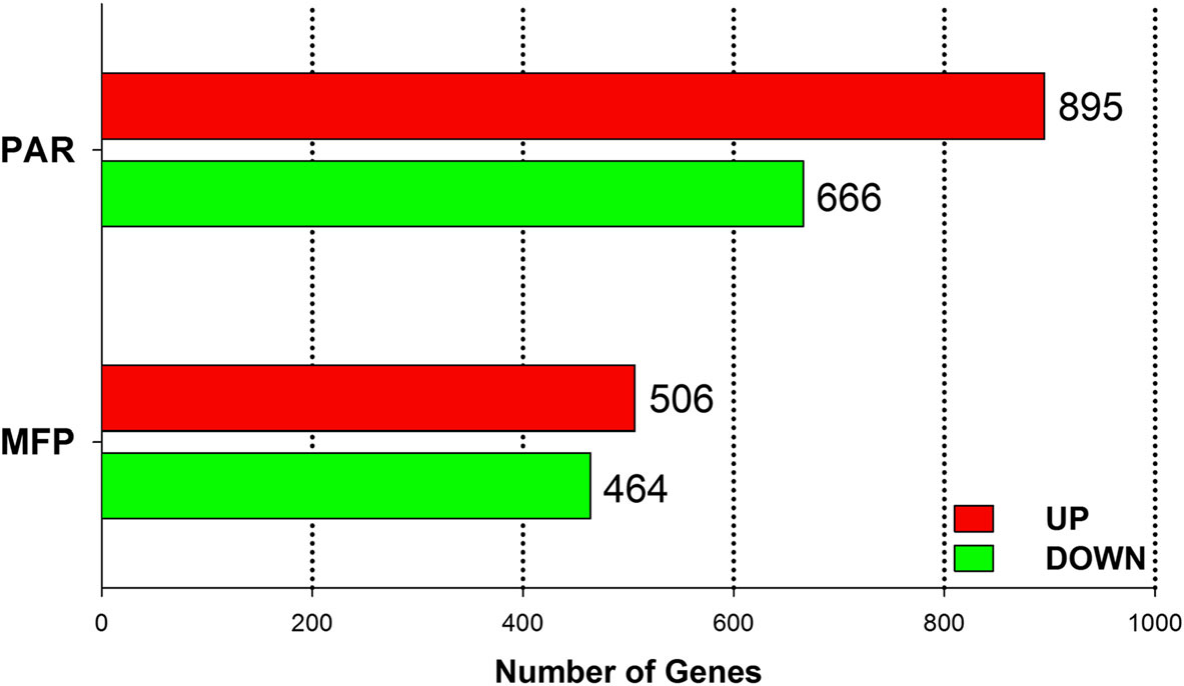
Differentially expressed genes (FDR < 0.05) detected in mammary parenchyma (PAR) and fat pad (MFP) of Holstein heifer calves (*n* = 6/treatment) fed pre-weaning (week 1 to 8 of life) an enhanced (EH) (1.08 kg of powder/calf/day, 28.9% crude protein, 26.2% fat, DM basis) or restricted (R; represents the industry standard or control) milk replacer (0.44 kg of powder/head/day, 20.9% crude protein, 19.8% fat, DM basis)

A summary of sequencing read alignments and mapping is reported in Additional file 2. Overall, on average, samples had approximately 27 million reads of which 25 million (~ 92%) were uniquely mapped. The statistical analysis identified 15,214 and 14,223 uniquely annotated (EntrezID) genes in PAR and MFP, respectively. Of these, considering an FDR < 0.05, 1561 genes (upregulated 895, downregulated 666) and 970 genes (upregulated 506, downregulated 464) were detected as differentially expressed in PAR and MFP, respectively, when comparing EH to R calves (Fig. 1).

### KEGG pathway analysis summary

The DIA analysis yields the impact and flux of all the manually-curated pathways included in the KEGG database. The term “impact” refers to the biological importance of a given pathway as a function of the change in expression of genes composing the pathway (proportion of DEG and their magnitude) in response to a treatment, condition, or change in physiological state [31]. Consequently, the direction of the impact, or flux, characterizes the average change in expression as up-regulation/activation, down-regulation/inhibition, or no change.

**Fig. 2:**
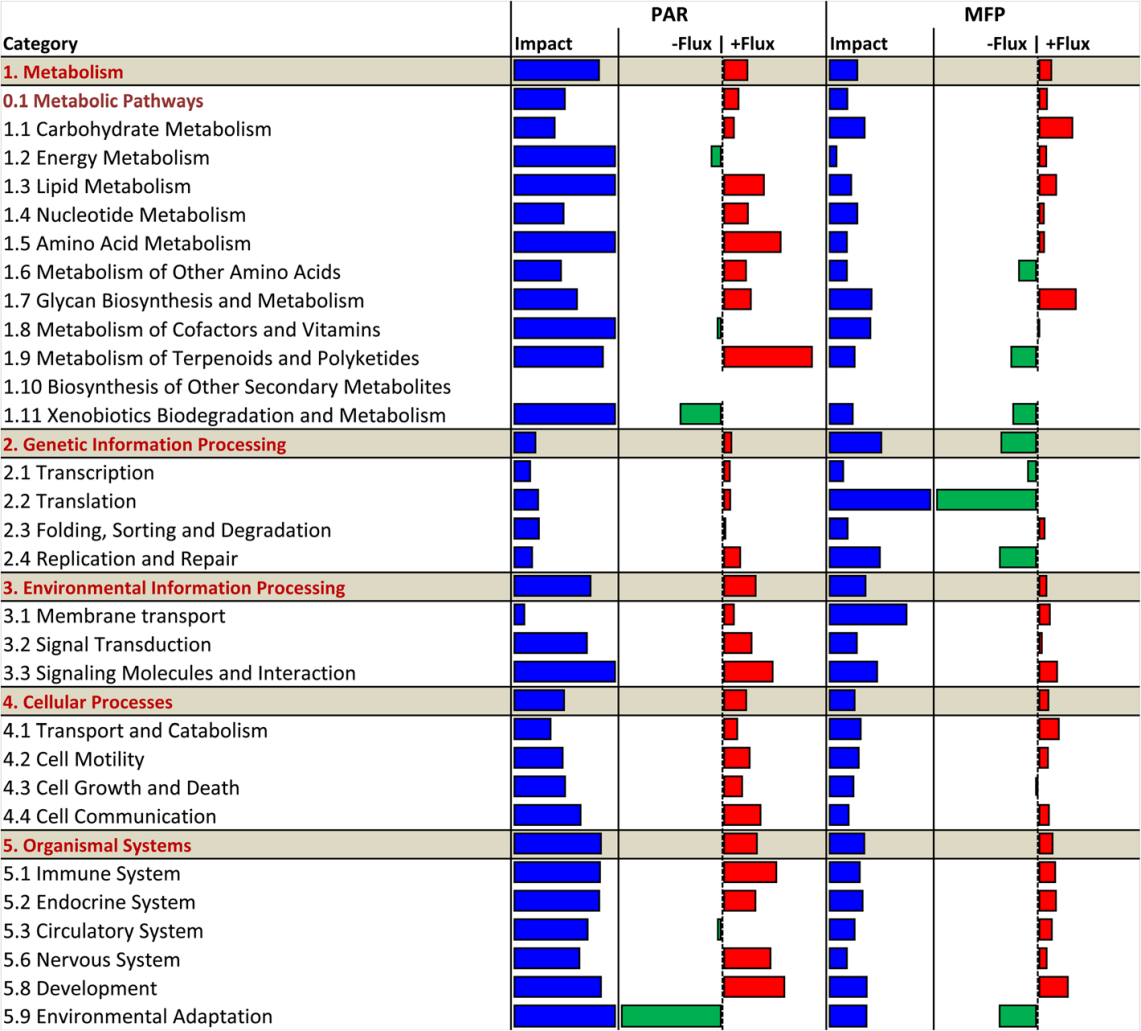
Summary of KEGG metabolic subcategories resulting from the DIA analysis in mammary parenchyma (PAR) or fat pad (MFP) of Holstein heifer calves (*n* = 6/treatment) fed pre-weaning (week 1 to 8 of life) an enhanced (EH) (1.08 kg of powder/head/day, 28.9% crude protein, 26.2% fat, DM basis) or restricted (R; represents the industry standard or control) milk replacer (0.44 kg of powder/calf/day, 20.9% crude protein, 19.8% fat, DM basis). For each tissue, the columns represent the effect (impact) and flux responses. The blue bars represent the effect value (0 to 30), and the flux columns represent negative (−) and positive (+) flux (−30 to + 30) based on the direction of the effect. The positive flux (green bars) indicates an upregulation in EH-fed heifer calves, while the negative flux (red bars) indicates an upregulation in R-fed heifer calves

Feeding an accelerated (EH) compared to a restricted (R) milk replacer pre-weaning had broad effects on the transcriptome (Fig. 2). All categories, both metabolic (‘Metabolism’) and non-metabolic (‘Genetic information processing’, ‘Environmental information processing’, ‘Cellular processes’, and ‘Organismal systems’), were impacted and up-regulated (positive flux) in PAR of EH vs R calves, with greatest changes in ‘Metabolism’, ‘Environmental information processing’, and ‘Organismal systems’. Similar changes were observed in the MFP response to pre-weaning plane of nutrition; however, ‘Genetic information processing’ was the most impacted category, and contrary to PAR, it was down-regulated (negative flux) in EH vs R calves. Furthermore, despite the ‘Metabolism’ category having a general activation (up-regulation) trend in both tissues, tissue-specific differences could be detected in the fluxes of its subcategories: ‘Energy metabolism’ (downregulated PAR, upregulated MFP), ‘Metabolism of other amino acids’ (upregulated PAR, downregulated MFP) and ‘Metabolism of terpenoids and polyketides’ (upregulated PAR, downregulated MFP). Concerning ‘Metabolism of cofactors and vitamins’, despite different fluxes between the tissues (downregulated PAR, upregulated MFP), these were very close to zero, thus, suggesting the lack of a clear up- or down-regulation in both tissues.

### KEGG most impacted pathways and IPA functions

#### Mammary parenchyma

**Fig. 3:**
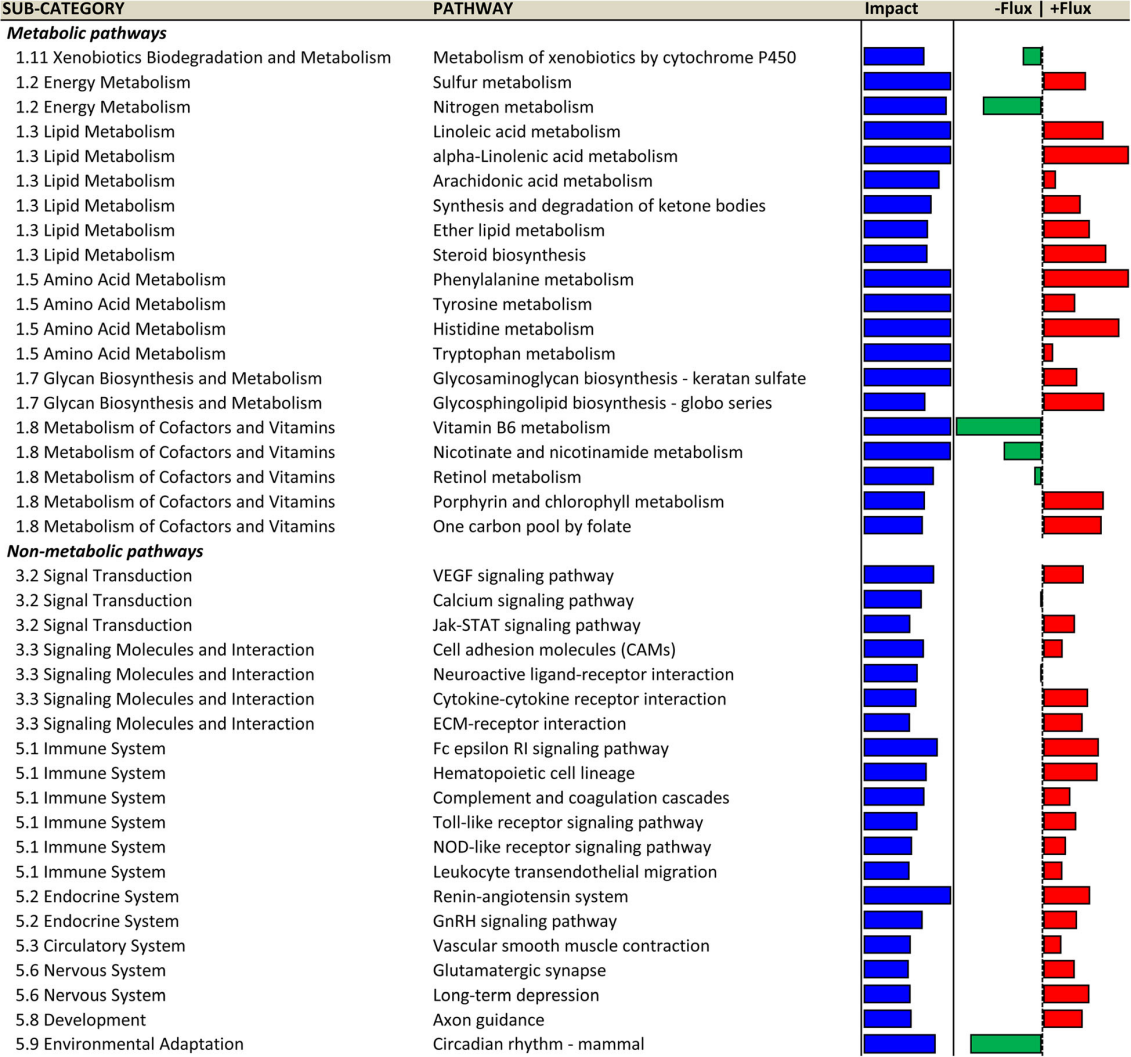
Dynamic Impact Approach (DIA) results (Impact and Direction of the Impact) for the most impacted metabolic and non-metabolic KEGG pathways (Top 20), grouped by sub-categories of pathways, in mammary parenchyma of Holstein heifer calves (n = 6/treatment) fed pre-weaning (week 1 to 8 of life) an enhanced (EH) (1.08 kg of powder/head/day, 28.9% crude protein, 26.2% fat, DM basis) or restricted (R; represents the industry standard or control) milk replacer (0.44 kg of powder/calf/day, 20.9% crude protein, 19.8% fat, DM basis). Blue bars represent the Impact (0–50) of dietary treatment, while red or green bars show the Direction of the Impact (−50 − + 50) (red = upregulation, green = downregulation, in EH vs R)

The top PAR metabolic and non-metabolic pathways are reported in Fig. 3, while the complete dataset of results is available in Additional file 3. The top-20 impacted metabolic pathways are included in the sub-categories ‘Amino acid metabolism’, ‘Energy metabolism’, ‘Glycan biosynthesis and metabolism’, ‘Lipid metabolism’, ‘Metabolism of Cofactors and Vitamins’, and ‘Xenobiotics biodegradation and metabolism’. Among these, all but 5 pathways were up-regulated in EH vs R: ‘Metabolism of xenobiotics by cytochrome P450’, ‘Nicotinate and nicotinamide metabolism’, ‘Nitrogen metabolism’, ‘Retinol metabolism’, and ‘Vitamin B6 metabolism’. Regarding the top-20 impacted non-metabolic pathways, they include the sub-category ‘Circulatory system’, ‘Development’, ‘Endocrine system’, ‘Environmental adaptation’, ‘Immune system’, ‘Nervous system’, ‘Signaling molecules and interaction’, ‘Signal transduction’. All but 3 pathways were up-regulated in EH vs R: ‘Circadian rhythm – mammal’ was down-regulated, while ‘Calcium signaling pathway’ and ‘Neuroactive ligand-receptor interaction’ had null flux, meaning they are biologically important during the pre-weaning physiological period despite plane of nutrition affecting the direction of flux.

**Fig. 4:**
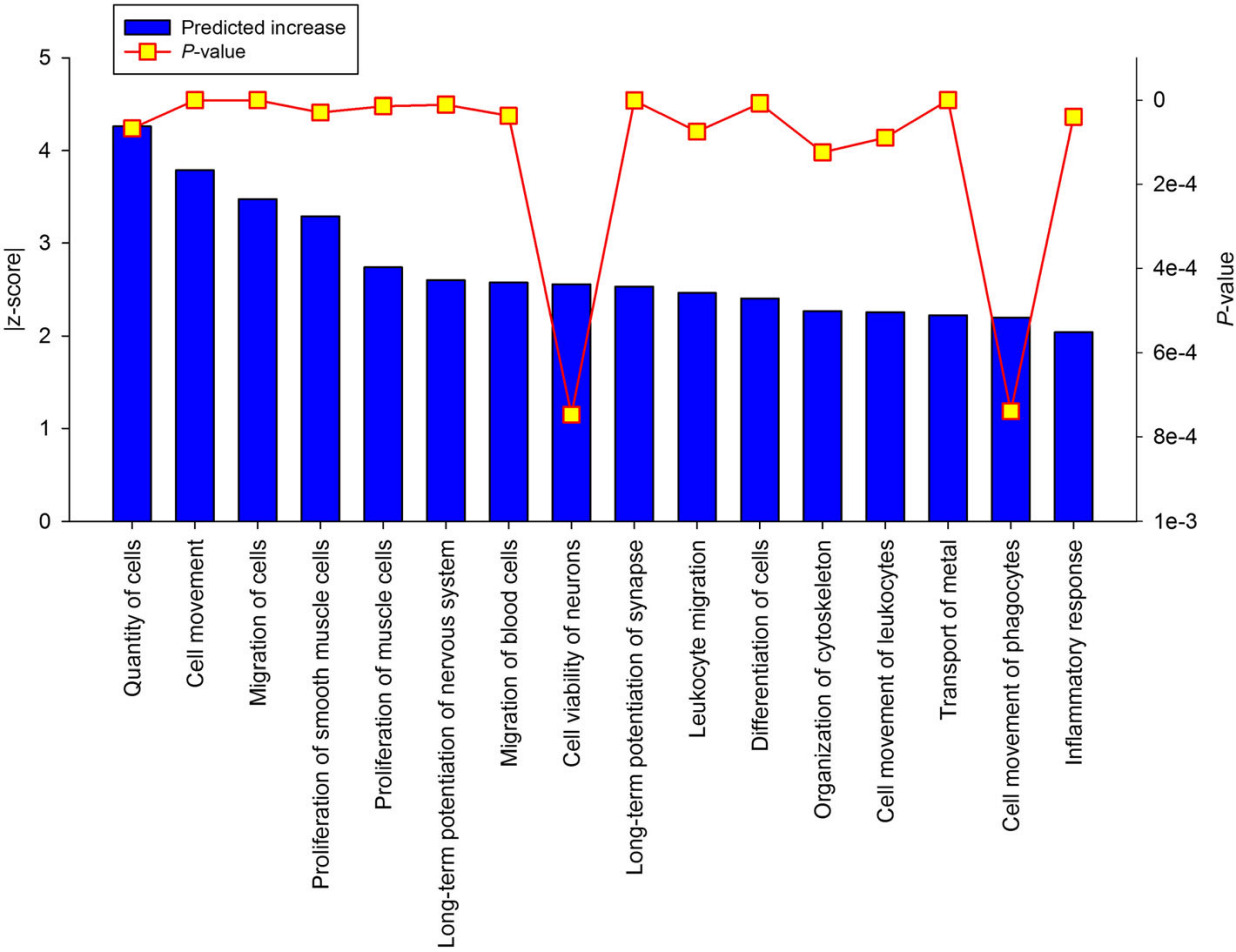
Ingenuity pathway analysis predicted canonical functions in mammary parenchyma of Holstein heifer calves (n = 6/treatment) fed pre-weaning (week 1 to 8 of life) an enhanced (EH) (1.08 kg of powder/calf/day, 28.9% crude protein, 26.2% fat, DM basis) or restricted (R; represents the industry standard or control) milk replacer (0.44 kg of powder/head/day, 20.9% crude protein, 19.8% fat, DM basis).

The IPA analysis identified 16 functions as significantly affected by the pre-weaning plane of nutrition, all predicted to be increased in EH rather than R calves. They encompass activities related to cell proliferation, differentiation, and growth, as well as the involvement of the immune system in PAR pre-pubertal development (Fig. 4).

#### Mammary fat pad

**Fig. 5:**
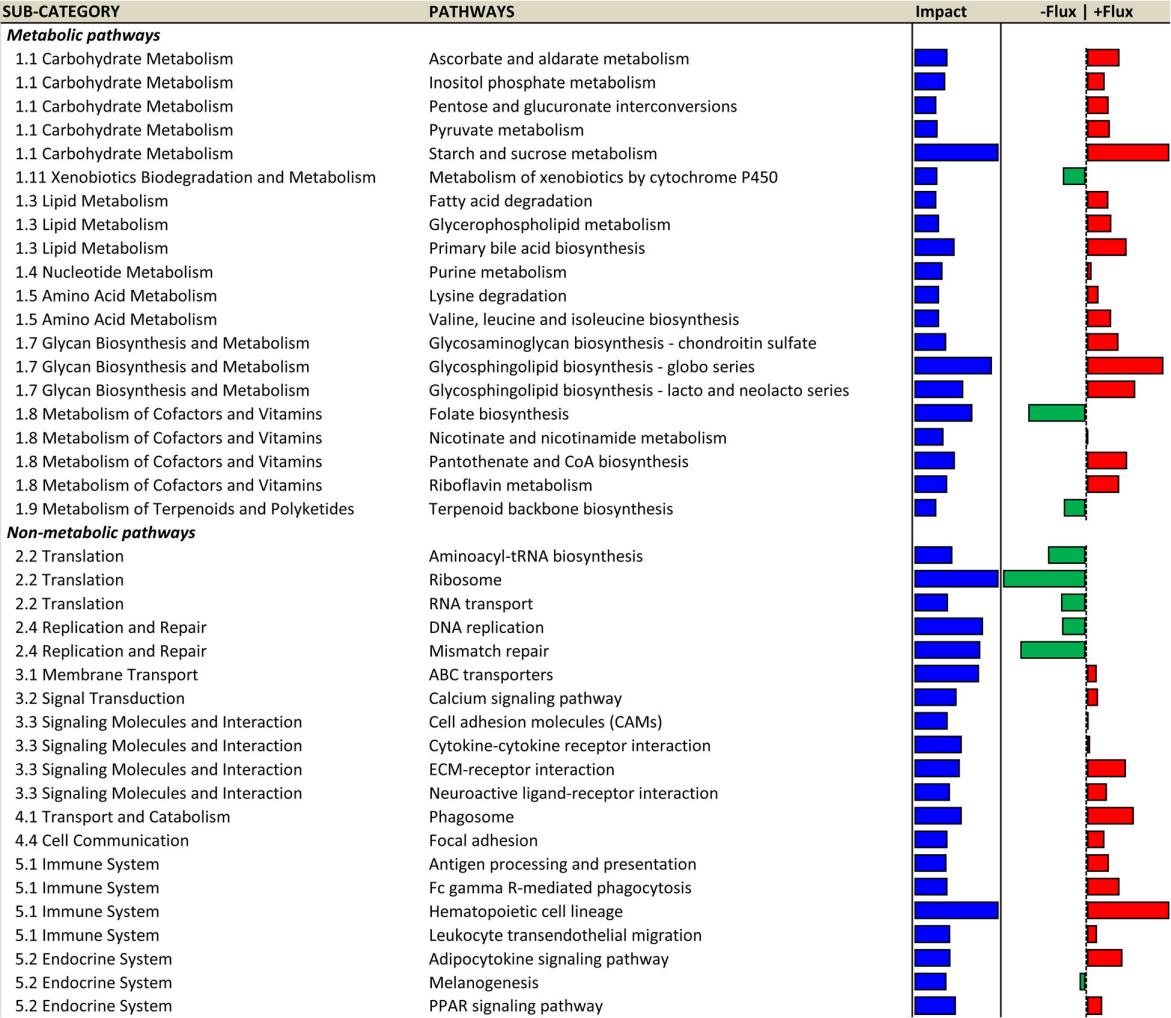
Dynamic Impact Approach (DIA) results (Impact and Direction of the Impact) for the most impacted metabolic and non-metabolic KEGG pathways (Top 20), grouped by sub-categories of pathways, in mammary fat pad of Holstein heifer calves (n = 6/treatment) fed pre-weaning (week 1 to 8 of life) an enhanced (EH) (1.08 kg of powder/head/day, 28.9% crude protein, 26.2% fat, DM basis) or restricted (R; represents the industry standard or control) milk replacer (0.44 kg of powder/calf/day, 20.9% crude protein, 19.8% fat, DM basis). Lines represent the Impact (0–30) of dietary treatment, while red or green bars show the Direction of the Impact (− 30 − + 30) (red = upregulation, green = downregulation, in EH vs R)

The top MFP metabolic and non-metabolic pathways are reported in Fig. 5, while the complete dataset of results is available in Additional file 3. The top-20 metabolic pathways are represented in the categories ‘Amino acid metabolism’, ‘Carbohydrate metabolism’, ‘Glycan biosynthesis and metabolism’, ‘Lipid metabolism’, ‘Metabolism of cofactors and vitamins’, ‘Metabolism of terpenoids and polyketides’, ‘Nucleotide metabolism’, and ‘Xenobiotics biodegradation and metabolism’. All but 4 pathways were up-regulated in EH vs R: ‘Folate biosynthesis’, Metabolism of xenobiotics by cytochrome p450’, and ‘Terpenoid backbone biosynthesis’ were down-regulated, while ‘Nicotinate and nicotinamide metabolism’ had a null flux. Among the top-20 non metabolic pathways, the sub-categories were ‘Cell communication’, ‘Endocrine system’, ‘Immune system’, ‘Membrane transport’, ‘Replication and repair’, ‘Signal transduction’, ‘Signal molecules and interaction’, ‘Translation’, and ‘Transport and catabolism’. All but 7 of these pathways were up-regulated in EH vs R: ‘Aminoacyl-tRNA biosynthesis’, ‘DNA replication’, ‘Melanogenesis’, ‘Mismatch repair’, ‘Ribosome’, and ‘RNA transport’ were down-regulated, while ‘Cell adhesion molecules (CAMs)’ had a null flux.

**Fig. 6:**
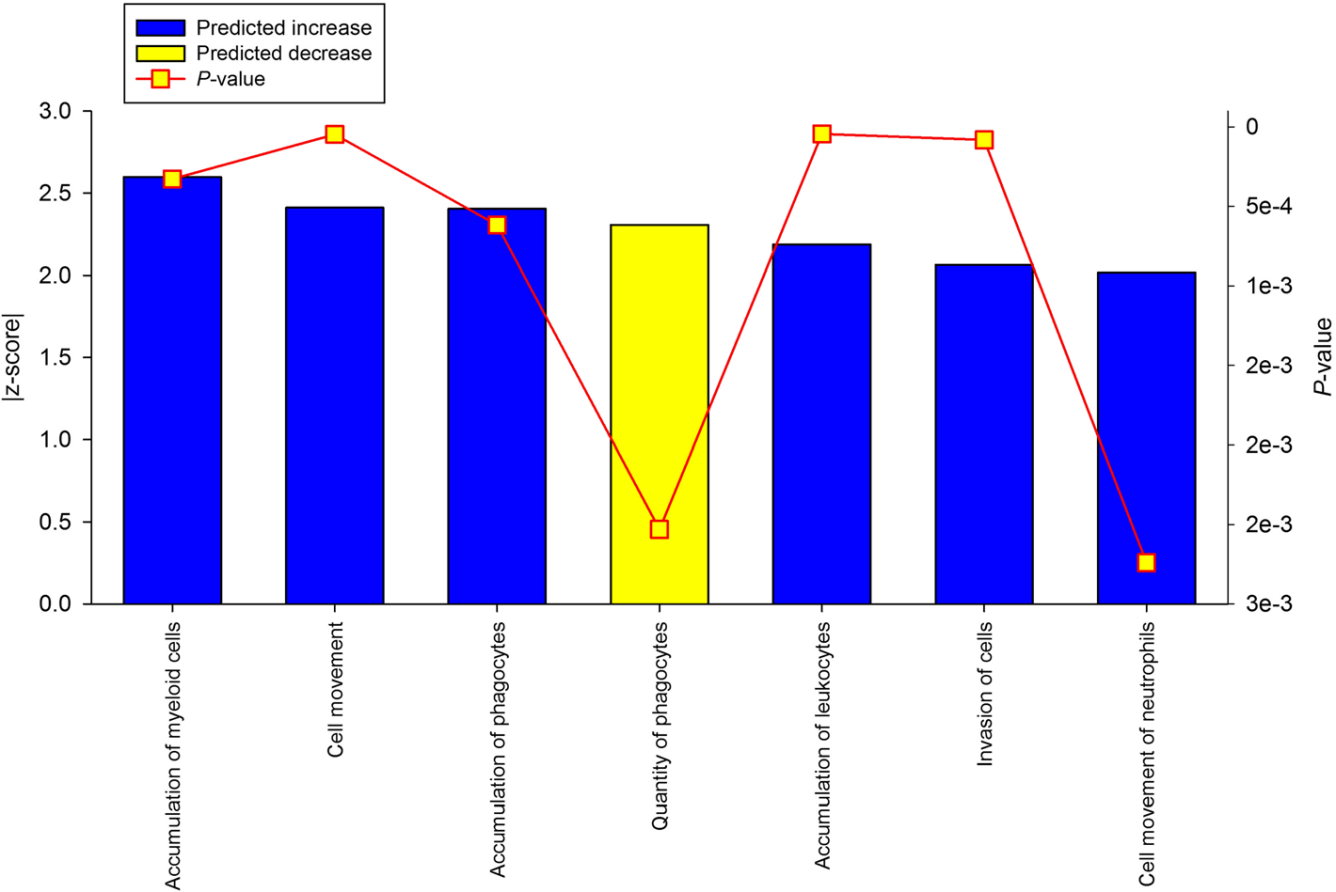
Ingenuity pathway analysis predicted canonical functions in mammary fat pad of Holstein heifer calves (n = 6/treatment) fed pre-weaning (week 1 to 8 of life) an enhanced (EH) (1.08 kg of powder/calf/day, 28.9% crude protein, 26.2% fat, DM basis) or restricted (R; represents the industry standard or control) milk replacer (0.44 kg of powder/head/day, 20.9% crude protein, 19.8% fat, DM basis)

The IPA analysis identified 7 significant functions, 6 predicted to be increased and 1 predicted to be decreased. All the functions were related to immune cell trafficking, invasion, and accumulation (Fig. 6).

### Up-stream regulators

The upstream regulator analysis generated 1122 and 685 possible regulators in PAR and MFP, respectively. Of these, only 69 (PAR; 58 predicted activated and 11 predicted inhibited) and 32 (MFP; 28 predicted activated and 4 predicted inhibited) were considered significantly involved in the response to plane of nutrition in EH vs R calves. In MFP, 10 (8 predicted activated, 2 predicted inhibited) of the significant upstream regulator were considered biased by IPA due to the skewness of the down-regulated genes toward up- or down-regulation. The full list of upstream regulators is reported in Tables 3 and 4 for PAR and MFP, respectively. Overall, the predicted regulators in both tissues were part of the molecule categories ‘chemical – endogenous mammalian’, ‘complex’, ‘cytokine’, ‘group’, ‘growth factor’, ‘ligand-dependent nuclear receptor’, ‘transcription regulator’, ‘transmembrane receptor’, and ‘others’. Unique to PAR were the categories ‘kinase’, ‘mature microrna’, and ‘microrna’, while ‘peptidase’, ‘phosphatase’, and ‘transporter’ were unique to MFP.

**Table 3:** IPA-predicted up-stream regulators in mammary parenchyma (PAR) of Holstein heifer calves (*n* = 6/treatment) fed pre-weaning (week 1 to 8 of life) an enhanced (EH) (1.08 kg of powder/calf/day, 28.9% crude protein, 26.2% fat, DM basis) or restricted (R; represents the industry standard or control) milk replacer (0.44 kg of powder/head/day, 20.9% crude protein, 19.8% fat, DM basis)

Upstream Regulator	Fold Change	Molecule Type	Predicted State	Z-score	*P*-value	Target molecules in dataset
arachidonic acid		chemical - endogenous mammalian	Activated	2.177	0.0185	*AQP3,EGFR,EPHB1,HMOX1,SLC2A4*
hyaluronic acid		chemical - endogenous mammalian	Activated	2.176	0.0406	*CXCL10,MMP2,NCF2,PTHLH,SERPINE1*
sphingosine-1-phosphate		chemical - endogenous mammalian	Activated	2.219	0.0141	*ANGPT1,CXCL10,EGFR,HAS2,SERPINE1*
tretinoin		chemical - endogenous mammalian	Activated	2.45	0.000000758	*ABHD2,ALDH1A3,APBB1IP,APLN,B3GALT5,BPI,BRCA1,CCND2,CCNE1,CCR1,CSF3R,CXCL10,CYP1A1,DAPK2,DHRS9,EGFR,FABP5,FOLR2,GFAP,GJA1,GPR21,HAS2,HEY2,HMOX1,HOXD1,HSD17B11,LIPA,MAFB,MAPK10,MMP19,MSC,MSI1,MTHFD2,NCF2,NEFH,PLEK,PLP1,POSTN,PTGIS,PTHLH,RAI14,SEMA5B,SERPINE1,SLC2A4,STRA6,Stra6l,STRA8,TP73,TRPC4,ZP2*
CD3		complex	Inhibited	−2.605	0.0126	*AQP3,C2,CCND2,CCNE1,CCR1,CD5,CD8B,CDC25A,CXCL10,EEF1A2,EPHB1,IL15RA,MAF,MMP2,MTHFD2,PLEK,TFEC,TFPI2,TP73*
LDL		complex	Activated	2.175	0.0475	*CXCL10,HAS2,HMOX1,IL1RN,MMP2,MSC,RYR2,SERPINE1*
NFkB (complex)		complex	Activated	2.378	0.000618	*ADAMTS9,ALDH1A3,CCND2,CXCL10,CXCL5,CXCL9,DBP,DIO1,EGFR,FABP5,GFAP,HAS2,HMOX1,IL15RA,IL1RN,MMP2,NCF2,NR1D1,SERPINE1,SLC2A4,TFEC,TFPI2*
Pdgf (complex)		complex	Activated	2.36	0.0000339	*CCND2,EGFR,GJA1,HEY2,HMOX1,MEOX2,MMP2,PIK3R3,SERPINE1*
PDGF BB		complex	Activated	2.851	0.000116	*ACAT2,BRCA1,CCNE1,CNN1,EGFR,FBLN5,FOXC2,FOXS1,GDA,GJA1,HMOX1,MMP2,MYH11,POSTN,SERPINE1,VCAN*
PI3K (complex)		complex	Activated	3.227	0.0000982	*ADAM12,APLN,CCND2,CCNE1,CCR1,CXCL10,FABP5,GJA1,HMOX1,IL1RN,MMP2,MTHFD2,PLA2G5,POSTN,SERPINE1*
Pka		complex	Activated	2.213	0.0267	*CXCL10,GJA1,HAS2,HMOX1,PTHLH,SLC1A3*
CSF2		cytokine	Activated	2.644	0.0101	*ACE,BUB1B,CCR1,CXCL10,ERCC6L,FIGNL1,FOLR2,HAS2,IL1RN,MME,MMP2,NLRP3,POLE,TARP,UBD*
CSF3	1.023	cytokine	Activated	2.393	0.000635	*CCND2,CCNE1,CSF3R,CXCL5,GFAP,GJA1,HMOX1,IL1RN,LYZ,MMP2*
EDN1	−1.29	cytokine	Activated	2.599	0.0021	*ADAM12,ADAM19,APLN,CDC25A,EGFR,GJA1,MMP2,RAMP3,SERPINE1,VCAN*
IFNG		cytokine	Activated	2.381	0.0000278	*ACOD1,ADORA1,ALDH1A3,C2,CALB2,CCND2,CCR1,CXCL10,CXCL9,DBP,DIO1,FABP5,FAM107A,FCN1,GFAP,GJA1,GPR146,HAS2,HCK,HMOX1,IL15RA,IL1RN,MMP2,NCF2,NDRG4,NLRP3,NR1D1,NTF4,PIGR,PLA2G5,PLEK,PTHLH,SERPINE1,SLC11A1,SLC1A3,SLC6A1,SYNM,TMOD1,TP73,TREM2,TRPC4,UBD*
IL1A		cytokine	Activated	2.794	0.0123	*ALDH1A3,CXCL10,CXCL5,CYP1A1,HMOX1,IL1R2,IL1RN,MMP2,SERPINE1*
IL1B	1.404	cytokine	Activated	2.1	0.00000379	*ACOD1,ANGPT1,CCR1,CXCL10,CXCL5,CXCL9,CYP1A1,DBP,DIO1,FABP5,GJA1,HAS2,HMOX1,IL15RA,IL1R2,IL1RN,LHB,MAPT,MMP2,MYH11,NR1D1,PIGR,PLA2G5,POSTN,PTGIS,PTHLH,SEMA3A,SERPINE1,SLC1A3,SLC2A4,TFPI2,TREM2,UBD,VCAN*
IL27		cytokine	Activated	2.747	0.00154	*CCND2,CXCL10,CXCL9,HPGD,IL15RA,IL1RN,MAF,UBD*
IL4		cytokine	Activated	2.113	0.00411	*ADAM19,CCND2,CCNE1,CD5,CSF3R,CXCL10,CXCL9,GJA1,HCK,HPGD,IL15RA,IL1R2,IL1RN,MAF,MAFB,MAL,MMP2,PIGR,PLOD2,POSTN,PTHLH,SERPINE1,TFEC,TREM2*
SPP1	1.148	cytokine	Activated	2.724	0.0000515	*ANGPT1,BRCA1,CCNE1,CDC25A,CXCL5,EGFR,GFAP,HAS2,HMOX1,MMP2,S100A4,SERPINE1*
TNF	1.151	cytokine	Activated	3.185	0.00000109	*ACE,ACOD1,ADAMTS7,ADORA1,ALDH1A3,ANGPT1,APLN,AQP3,BMPER,BUB1B,CCND2,CCR1,CD5,CDH11,CNN1,CXCL10,CXCL5,CXCL9,CYP1A1,DIO1,EGFR,ENTPD5,FABP5,FOXC2,GFAP,GJA1,HMOX1,HPGD,IL15RA,IL1R2,IL1RN,MEOX2,MMP2,MSC,NCF2,NEFH,NLRP3,PDE2A,PER2,PIGR,PLA2G5,PLOD2,PLP1,POSTN,PPP1R3C,PTHLH,SERPINE1,SLC11A1,SLC1A3,SLC2A4,SORBS1,TFPI2,TREM2,UBD,WISP1*
TNFSF12	−1.07	cytokine	Activated	2.163	0.0193	*ADAM12,CCR1,CXCL10,IL1R2,SLC2A4*
Akt		group	Activated	2.521	0.000711	*ANGPT1,AQP3,BRCA1,CCND2,CXCL10,EGFR,HMOX1,HPGD,MMP2,SERPINE1,SLC2A4,VCAN*
Alpha catenin		group	Inhibited	−2.433	0.0159	*ADAMTS12,CDH11,CXCL10,LYZ,MMP19,MMP2*
ERK		group	Activated	2.636	0.000184	*CCNE1,CCR1,CXCL10,CYP1A1,EGFR,HEY2,HMOX1,MAFB,MAL,MMP2,SERPINE1,STMN4,VCAN*
ERK1/2		group	Activated	3.053	0.000325	*APLN,BRCA1,CCND2,CDC25A,CXCL10,CXCL5,GJA1,HAS2,HPGD,MMP2,POSTN,SERPINE1,VCAN,WISP1*
IL1		group	Activated	2.622	0.0000596	*C2,CXCL10,CXCL5,CXCL9,CYP1A1,DDC,EGFR,GFAP,GJA1,HMOX1,IL1R2,IL1RN,LYZ,MMP2,PIGR,SERPINE1,VCAN*
Jnk		group	Activated	2.211	0.00202	*APLN,BRCA1,CCNE1,GJA1,HMOX1,LHB,MMP2,POSTN,SERPINE1,VCAN*
Mek		group	Activated	2.602	0.0164	*CCND2,CXCL10,CXCL9,GDA,HAS2,LHB,SERPINE1,SLC16A6*
Nr1h		group	Inhibited	−2.183	0.000167	*ACE,AQP3,C2,CCND2,CXCL10,HCK,MPZ,NLRP3,SLC2A4,TRPC4*
P38 MAPK		group	Activated	2.164	0.00109	*ANGPT1,CCND2,CCNE1,CXCL10,CXCL9,CYP1A1,DDC,GJA1,HMOX1,IL1RN,MAL,MMP2,MYH11,POSTN,SERPINE1*
Tnf (family)		group	Activated	2.416	0.0341	*CXCL10,CXCL9,CYP1A1,GJA1,HTR2A,SERPINE1*
AGT		growth factor	Activated	2.57	0.00000192	*ACAT2,ACE,ADAM12,ANGPT1,APLN,BRCA1,CCND2,CCNE1,CDC25A,CXCL10,EGFR,FOXP2,GJA1,HAS2,HMOX1,MAPT,MMP2,NCF2,POSTN,PPP1R3C,PTHLH,SCN2A,SERPINE1*
ANGPT2	−1.107	growth factor	Activated	2.17	0.00155	*ADAM12,ASPHD1,EGFR,HMOX1,MAPK10,MAPK12,MMP2,POSTN,PTHLH,SERPINE1*
EGF		growth factor	Activated	2.738	0.00000409	*ANGPT1,AQP3,BRCA1,CCND2,CCNE1,CDH11,CXCL5,CYP1A1,E2F3,EGFR,GFAP,GJA1,HAS2,HMOX1,HPGD,IL1R2,MMP2,PLAGL1,PTHLH,S100A4,SERPINE1,SLC2A4,TFPI2,VCAN*
FGF2	−1.127	growth factor	Activated	2.287	0.000375	*ACE,ANGPT1,AQP3,CCND2,CDC25A,CDH11,EGFR,GFAP,GJA1,HAS2,MAPT,MEOX2,MMP2,MPZ,S100A4,SERPINE1*
INHA	1.185	growth factor	Inhibited	−2.067	0.0000109	*CCND2,CCNE1,CNN1,EGFR,HAS2,HSD17B11,MMP2,MYH11,SERPINE1,WISP1*
JAG2	−1.135	growth factor	Inhibited	−2	0.00417	*CCR1,CXCL5,CXCL9,IL1RN*
NRG1	−1.228	growth factor	Activated	2.252	0.0351	*ANGPT1,BRCA1,CCND2,CCNE1,GDA,HMOX1,PYGM,SLC2A4*
TGFB1	1.118	growth factor	Activated	2.435	1.64E-09	*ACE,ACKR1,ADAM12,ADAM19,ADAMTS12,ADORA1,ANGPT1,APLN,BUB1B,C2,CALB2,CCND2,CCNE1,CCR1,CDC25A,CDH11,CLCA2,CNN1,COL11A1,CPXM1,CRHR2,CRMP1,CXCL10,FABP5,FAM107A,FBLN5,FCER1A,FOXC2,GFAP,GJA1,GPR146,GPR21,HAS2,HMOX1,HOXD1,HPGD,IL1RN,KCNMB1,MAF,MMP2,MPZ,MTHFD2,MYH11,NCF2,NDRG4,NLRP3,PLAGL1,PLOD2,PLXNC1,POSTN,PPP1R3C,PRKCG,PTHLH,S100A4,SEMA3A,SERPINE1,TENM4,TP73,USH1C,VCAN,WISP1,XPNPEP2*
TGFB2	1.073	growth factor	Activated	2.184	0.000322	*CDH11,CNN1,HAS2,HMOX1,PLOD2,PTHLH,SERPINE1,VCAN*
AKT1	−1.008	kinase	Activated	2.424	0.0000153	*ACAT2,CCND2,CCNE1,CXCL10,DLX3,FABP5,HMOX1,LAMA1,LIPA,MMP2,MYH11,PLP1,RAMP3,SERPINE1,VCAN*
AR		ligand-dependent nuclear receptor	Activated	2.362	0.0000416	*AQP3,BUB1B,CA4,CDH11,EGFR,FABP5,GFAP,GJA1,LMOD1,MME,MSI1,MTHFD2,NTF4,PIGR,Pln,PLOD2,PTHLH,SERPINE1,TARP,VCAN*
miR-16-5p (and other miRNAs w/seed AGCAGCA)		mature microrna	Inhibited	−2.185	0.021	*CCND2,CCNE1,CDC25A,CHEK1,E2F3,EGFR,HMOX1,PLAG1*
mir-155		microrna	Inhibited	−2.213	0.00404	*CXCL10,EGFR,HMOX1,MAF,MME,SERPINE1*
CAMP	4.064	other	Activated	2.22	0.0141	*CSF3R,CXCL10,CXCL5,IL1R2,IL1RN*
RETNLB		other	Activated	2	0.0417	*CCR1,LAMA1,MMP2,WISP1*
EGR1	−1.092	transcription regulator	Activated	2.217	0.000185	*ACE,CCND2,CCR1,EGFR,ENPEP,HMOX1,HPGD,LHB,LYZ,SERPINE1,TP73*
FOXM1	1.769	transcription regulator	Activated	2.405	0.0136	*BUB1B,CCND2,CCNE1,CDC25A,MMP2,VCAN*
MYC	1.009	transcription regulator	Activated	2.887	0.00135	*ACOD1,ANGPT1,BMPER,BRCA1,BUB1B,CCND2,CCNE1,CDC25A,CHEK1,CNTNAP2,CXCL10,DLX3,E2F3,FABP5,FBLN5,FBN2,FXYD1,GJA1,HAS2,HMOX1,KLK6,LYZ,NTF4,PCDH18,PLP1,PNCK,SCAMP5,SERPINE1,SLC11A1,TP73,WISP1*
NFKB1	−1.113	transcription regulator	Activated	2.369	0.00876	*ADORA1,CCND2,CRMP1,CXCL10,CXCL9,EGFR,GJA1,HAS2,HMOX1,IL1RN*
NOTCH1	1.053	transcription regulator	Activated	2.14	0.00536	*ADAM19,ANGPT1,CCND2,CCNE1,EGFR,GFAP,HEY2,MMP2,MPZ,SERPINE1*
SMAD2	1.071	transcription regulator	Activated	2.219	0.0128	*HAS2,HMOX1,MMP2,PTHLH,SERPINE1*
SMAD3	1.052	transcription regulator	Activated	2.184	0.0134	*ADORA1,CCND2,CCNE1,CXCL10,HAS2,HMOX1,LHB,PTHLH,SERPINE1*
SP1	1.148	transcription regulator	Activated	2.178	0.000139	*ABHD2,CCND2,CXCL10,CXCL5,DLX3,EGFR,FOLR2,GJA1,HAS2,HMOX1,LAMA1,LHB,LIPA,MEOX2,MMP2,MYH11,NCF2,NTF4,SERPINE1,SLC11A1,TFPI2,TP73*
SRF	−1.107	transcription regulator	Inhibited	−2.186	0.0248	*CNN1,CPM,FCN1,GDA,LMOD1,MAPK10,MAT1A,MYH11,PDE2A,RAI2,SEMA3A*
TLR2	1.392	transmembrane receptor	Activated	2.013	0.0183	*CCR1,CXCL10,GJA1,HMOX1,IL15RA,IL1RN,LHB*
TLR3	1.088	transmembrane receptor	Activated	2.183	0.0227	*ACOD1,CPM,CXCL10,HMOX1,IL1R2,IL1RN,LIPA,PIK3R3,SERPINE1*

**Table 4:** IPA predicted up-stream regulator in mammary fat pad (MFP) of Holstein heifer calves (n = 6/treatment) performances fed pre-weaning (week 1 to 8 of life) an enhanced (EH) (1.08 kg of powder/calf/day, 28.9% crude protein, 26.2% fat, DM basis) or restricted (R; represents the industry standard or control) milk replacer (0.44 kg of powder/head/day, 20.9% crude protein, 19.8% fat, DM basis)

Upstream Regulator	Fold Change	Molecule Type	Predicted State	Z-score	Flags	*P*-value	Target molecules in dataset
fatty acid		chemical - endogenous mammalian	Activated	2.186	bias	0.0000164	*APOA1,CYR61,ITGAM,NR4A3,PLIN4,S100A8*
tretinoin		chemical - endogenous mammalian	Activated	2.197		0.00000726	*ADM,ALPL,ANGPTL4,CLDN10,CPE,CYR61,EGR1,FFAR3,GPRC5A,ITGAM,LBP,MSC,NOV,PLA2G15,PTHLH,RET,RORC,S100A8,S100A9,SFRP1,SOCS1,TNFAIP6,WNT6*
LDL		complex	Activated	2.153		0.0000719	*EGR1,FCGR2B,IL1RN,MSC,NR4A3,S100A8,SOCS1,TNFAIP6*
PDGF BB		complex	Activated	2.775	bias	0.000559	*ADM,CYR61,EGR1,JUNB,LBP,NR4A3,TNFRSF12A,ZFP36*
CSF2		cytokine	Activated	2.255	bias	0.00128	*CD1A,CD1B,EGR1,FCGR2B,IL1RN,ITGAM,PLA2G15,SOCS1,ZFP36*
IL1B	−1.08	cytokine	Activated	2.13		0.000000111	*ADAMTS4,ADM,ALPL,ANGPTL4,BGN,EGR1,FCGR2B,IGFALS,IL1RN,ITGAM,JUNB,LBP,NR4A3,PTHLH,RORC,S100A8,S100A9,SOCS1,TNFAIP6,ZFP36*
IL2		cytokine	Activated	2.406	bias	0.0162	*ABCC1,FGF13,PTHLH,RGCC,RORC,S100A8,SOCS1,TNFRSF12A*
IL3		cytokine	Activated	2.63	bias	0.00329	*CD1A,EGR1,FCGR2B,ITGAM,JUNB,NOV,SOCS1*
IL5		cytokine	Activated	2.157	bias	0.0244	*CRELD2,EGR1,FCGR2B,ITGAM,SOCS1*
OSM		cytokine	Activated	2.303		0.0123	*ABCC1,ADAMTS4,JUNB,LBP,MSC,S100A8,S100A9,SOCS1*
TNF		cytokine	Activated	3.105		1.03E-09	*ABCC1,ADAMTS4,ADM,ANGPTL4,APOA1,ARSI,BGN,CD1A,CD1B,CYR61,EGR1,FCGR2B,IL17D,IL1RN,ITGAM,JUNB,LBP,LTBP2,MSC,NOV,NR4A3,PLIN4,PTHLH,RASSF7,RGCC,S100A8,S100A9,SFRP1,SNCG,SOCS1,TNFAIP6,ZFP36*
Alpha catenin		group	Inhibited	−3.11	bias	1.96E-09	*ADAMTS4,BGN,C1QTNF3,COL6A3,ITGAM,NOV,S100A8,S100A9,TNFAIP6,TNFRSF12A*
Creb		group	Activated	2.376		0.00034	*ADM,EGR1,GSTM3,ITGAM,JUNB,NR4A3,PTHLH,RET*
Pkc(s)		group	Activated	2.177		0.00022	*ADM,APOA1,CYR61,EGR1,JUNB,NR4A3,S100A8*
AGT		growth factor	Activated	2.064		0.00025	*ADM,BGN,CAV3,CGREF1,EGR1,FGF13,PTHLH,SOCS1,TNFRSF12A,ZFP36*
FGF2	1.31	growth factor	Activated	2.758		0.000229	*ANGPTL4,BGN,CYR61,EGR1,JUNB,NOV,S100A8,SFRP1,TNFRSF12A*
GH1		growth factor	Activated	2.046		0.0000304	*ABCC1,ANGPTL4,EGR1,IGFALS,JUNB,Pzp,SOCS1,ZFP36*
TGFB1	1.228	growth factor	Activated	2.393		0.00000222	*ADAMTS4,ADM,ANGPTL4,BGN,COL6A3,CPQ,CYR61,EGR1,GPRC5A,GUCY2C,IL17D,IL1RN,ITGAM,JUNB,LTBP2,NOV,NR4A3,P2RX6,PTHLH,RGCC,RORC,SFRP1,SOCS1,TNFAIP6,TNFRSF12A,ZFP36*
VEGFA	1.452	growth factor	Activated	2.354	bias	0.00413	*ABCC1,CD1A,CYR61,EGR1,JUNB,SNCG*
PPARG	1.209	ligand-dependent nuclear receptor	Activated	2.183		0.000332	*ADIPOR2,ANGPTL4,APOA1,GIPR,ME1,PLIN4,RORC,S100A8,SNCG,SOCS1*
S100A9	1.83	other	Activated	2.2		0.0000428	*ADAMTS4,ALPL,GPRC5A,ITGAM,NR4A3,S100A8,S100A9*
F2	1.281	peptidase	Activated	2.372	bias	0.00376	*ANGPTL4,CYR61,EGR1,JUNB,NR4A3,TNFRSF12A*
SOCS3	1.172	phosphatase	Inhibited	−2.219	bias	0.0000872	*EGR1,IGFALS,IL1RN,SGCA,SOCS1*
CREB1	1.047	transcription regulator	Activated	2.804		0.00127	*ADM,CGREF1,CYR61,EGR1,FGF13,JUNB,NR4A3,SRXN1,TP53INP2,ZFP36*
SMAD3	−1.022	transcription regulator	Activated	2.391		0.000318	*APOA1,BGN,COL6A3,EGR1,JUNB,PTHLH,ZFP36*
SMAD4	−1.014	transcription regulator	Activated	2.423		0.000262	*ANGPTL4,APOA1,BGN,PTHLH,RGCC,TNFAIP6,ZFP36*
SMAD7	1.072	transcription regulator	Inhibited	−2		0.00599	*BGN,CGREF1,COL6A3,LTBP2*
TP63		transcription regulator	Activated	2.159		0.0194	*ADM,CYR61,JUNB,PTHLH,S100A8,TINAGL1*
IL10RA	1.167	transmembrane receptor	Activated	2.236		0.0343	*CYR61,FADS3,IL1RN,NOV,TTR*
TREM1		transmembrane receptor	Activated	2		0.0286	*CD1A,EGR1,GIPR,GPRC5A*
SFTPA1		transporter	Inhibited	−2		0.000918	*ANGPTL4,CYR61,EGR1,GPR68*

### Tissue cross-talk

**Fig. 7:**
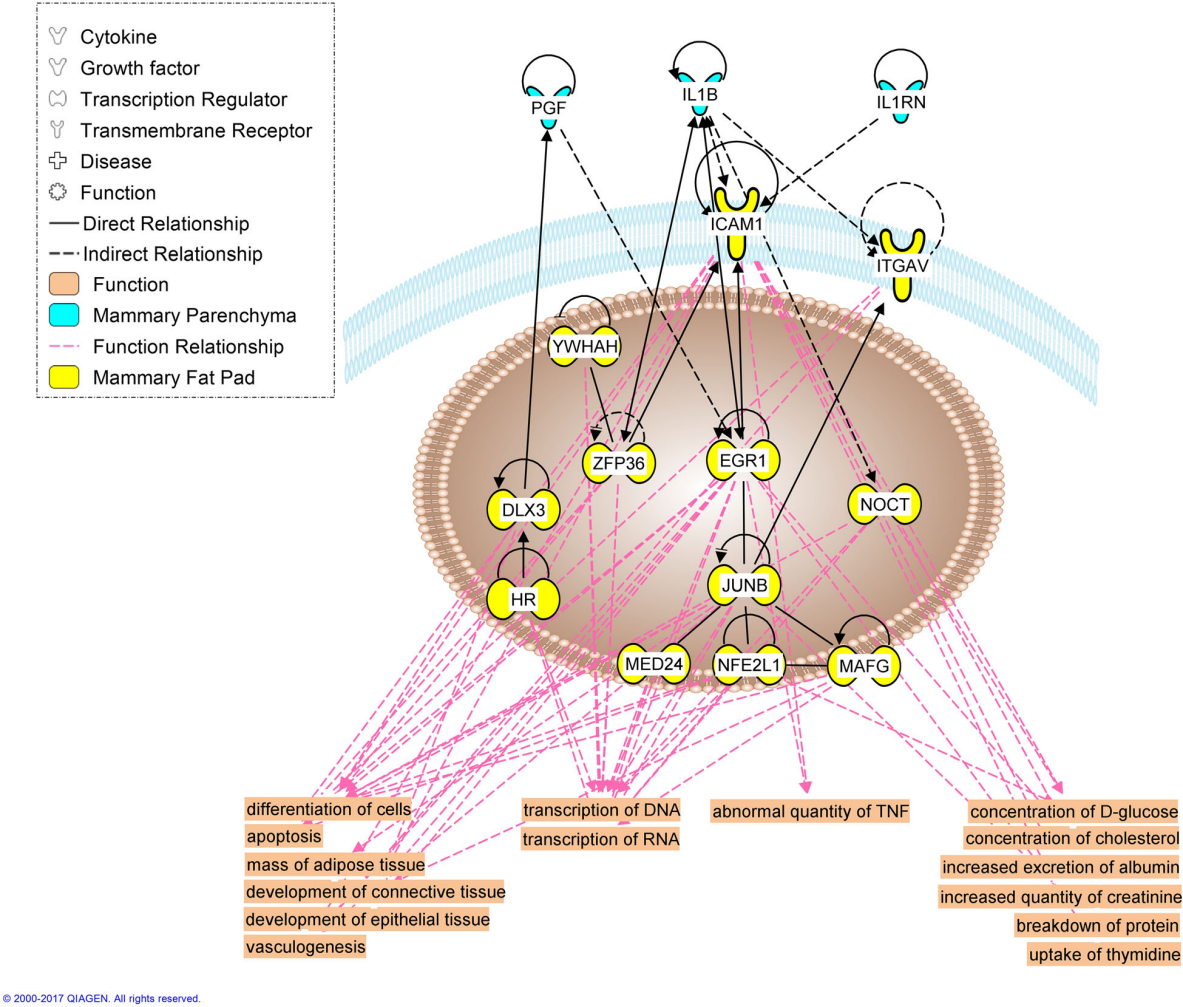
Role of mammary parenchyma (blue) in the tissue cross-talk with mammary fat pad (yellow) in Holstein heifer calves (n = 6/treatment) fed pre-weaning (week 1 to 8 of life) an enhanced (EH) (1.08 kg of powder/head/day, 28.9% crude protein, 26.2% fat, DM basis) or restricted (R; represents the industry standard or control) milk replacer (0.44 kg of powder/head/day, 20.9% crude protein, 19.8% fat, DM basis). The affected functions in the fat pad are highlighted at the bottom

**Fig. 8:**
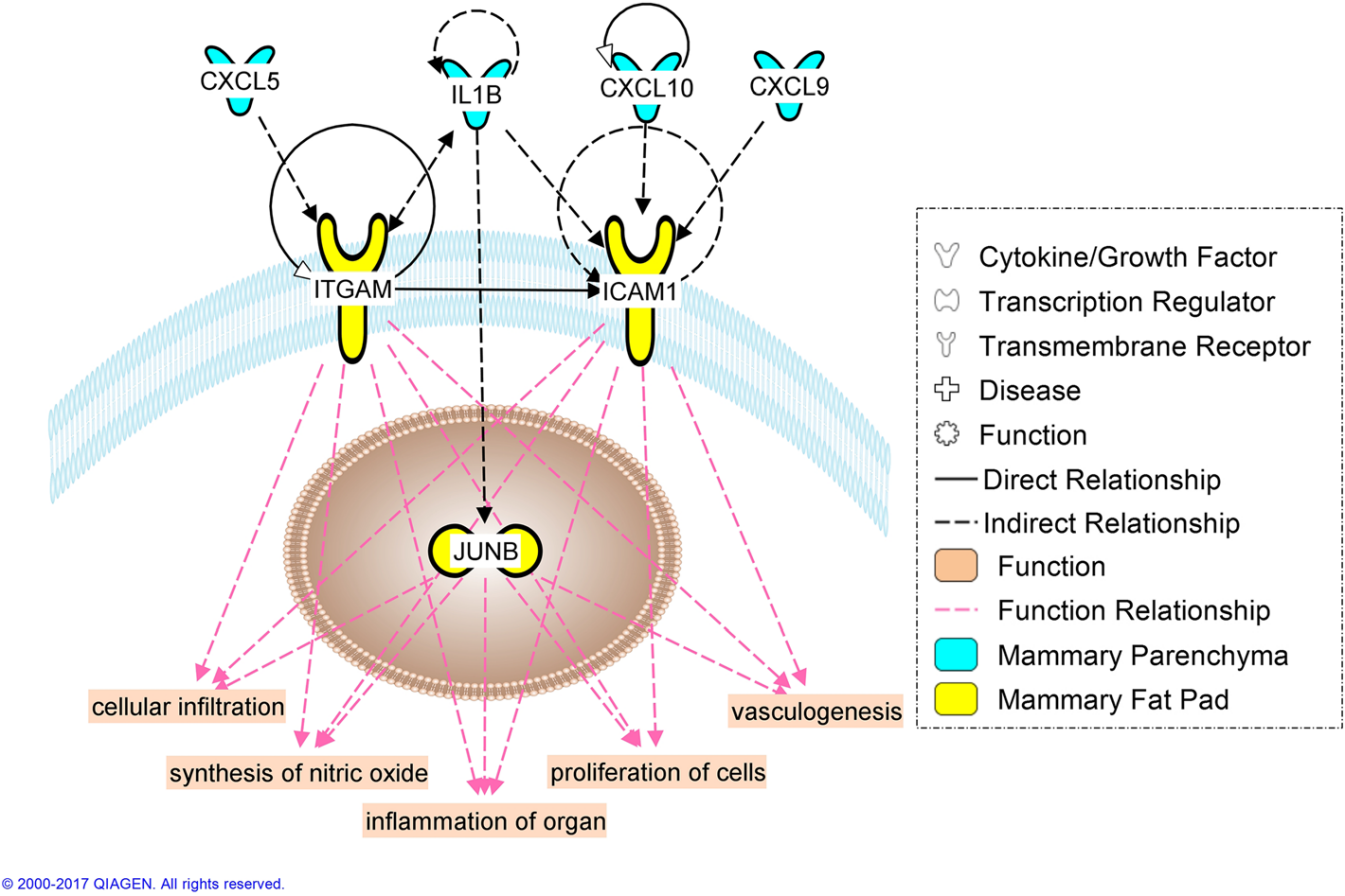
Involvement of the immune system in the tissue cross-talk between mammary parenchyma (blue) and mammary fat pad (yellow) in Holstein heifer calves (n = 6/treatment) fed pre-weaning (week 1 to 8 of life) an enhanced (EH) (1.08 kg of powder/head/day, 28.9% crude protein, 26.2% fat, DM basis) or restricted (R; represents the industry standard or control) milk replacer (0.44 kg of powder/head/day, 20.9% crude protein, 19.8% fat, DM basis). The affected functions in the fat pad are highlighted at the bottom

**Fig. 9:**
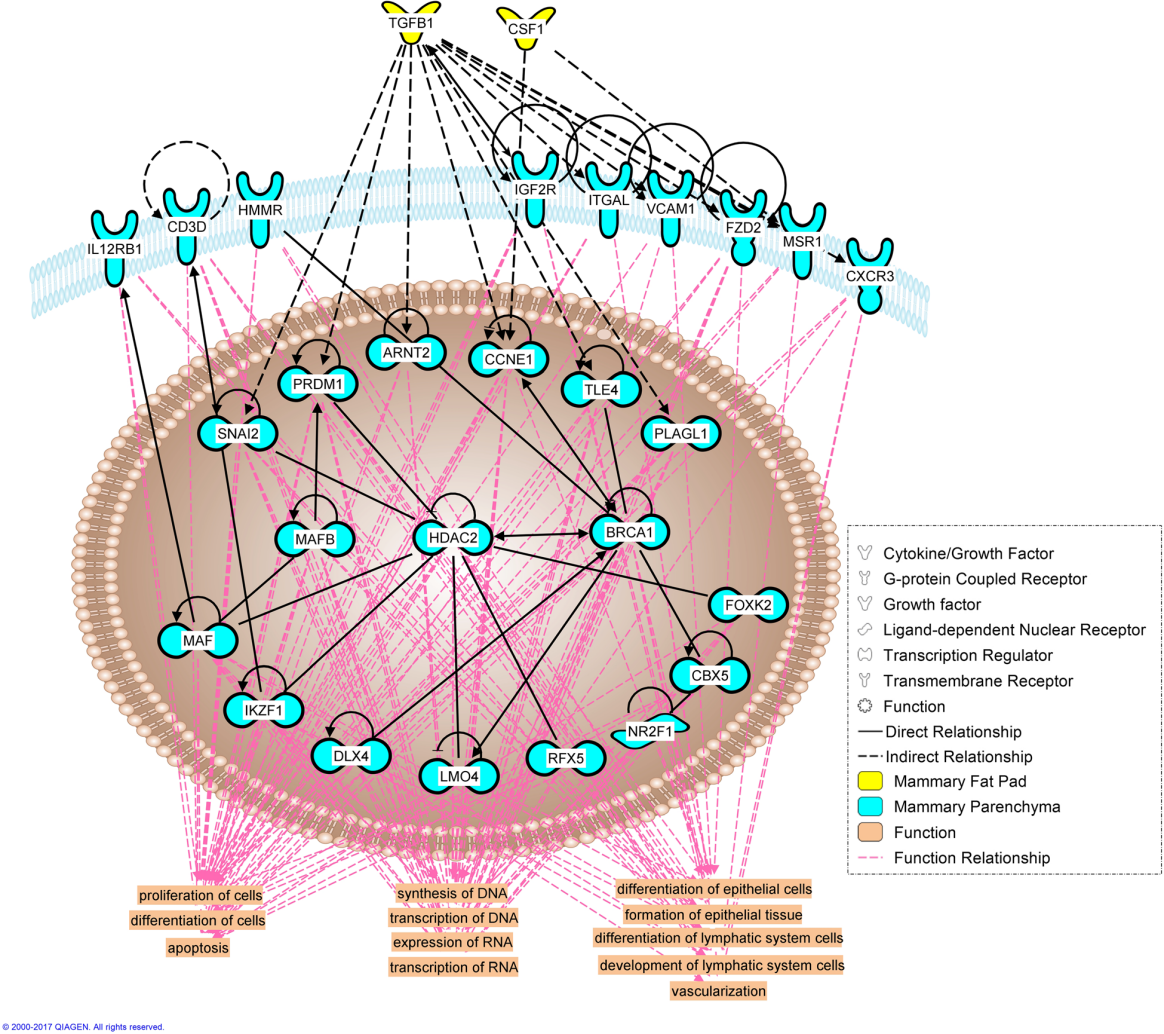
Role of mammary fat pad (yellow) in the tissue cross-talk with mammary parenchyma (blue) in Holstein heifer calves (n = 6/treatment) fed pre-weaning (week 1 to 8 of life) an enhanced (EH) (1.08 kg of powder/head/day, 28.9% crude protein, 26.2% fat, DM basis) or restricted (R; represents the industry standard or control) milk replacer (0.44 kg of powder/calf/day, 20.9% crude protein, 19.8% fat, DM basis). The affected functions in the parenchyma are highlighted at the bottom

**Fig. 10:**
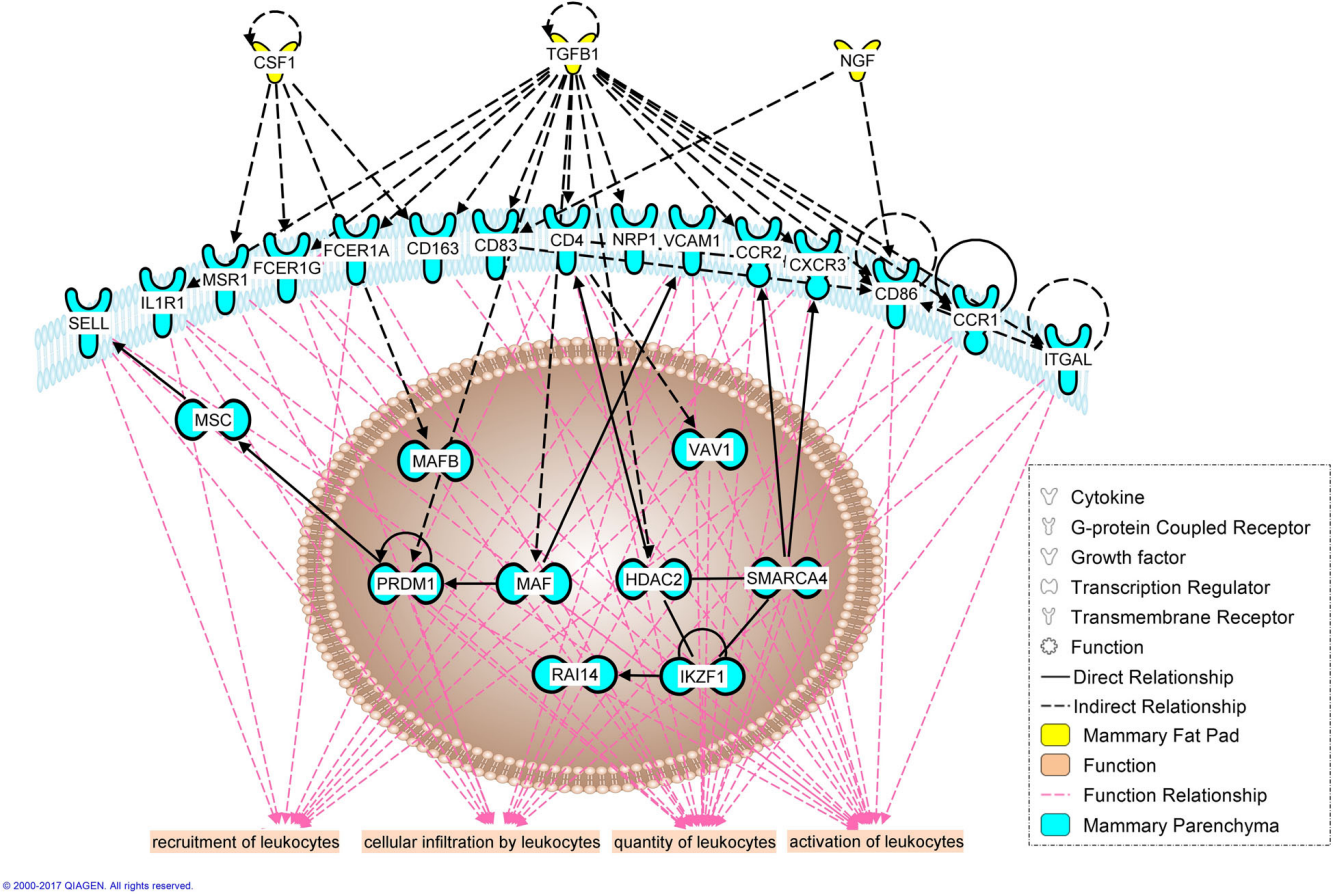
Involvement of the immune system in the tissue cross-talk between mammary fat pad (yellow) and mammary parenchyma (blue) in Holstein heifer calves (n = 6/treatment) fed pre-weaning (week 1 to 8 of life) an enhanced (EH) (1.08 kg of powder/calf/day, 28.9% crude protein, 26.2% fat, DM basis) or restricted (R; represents the industry standard or control) milk replacer (0.44 kg of powder/calf/day, 20.9% crude protein, 19.8% fat, DM basis). The affected functions in the parenchyma are highlighted at the bottom

Our analysis identified 14 and 8 up-regulated differentially expressed genes in EH vs R coding for secreted proteins, and 105 and 64 genes coding for possible receptors in PAR and MFP, respectively (Additional file 4). However, after automatic network construction with the IPA knowledge base, only some of these were deemed relevant to the cross-talk between these specific tissues. PAR differentially expressed (EH vs R) genes encoding 3 potentially secreted proteins (*PGF*, *IL1B*, *IL1RN*) which can influence MFP through 12 possible receptors differentially expressed by the latter (Fig. 7). In contrast, MFP differentially expressed genes encoding 2 potentially secreted proteins (*CSF1*, *TGFB1*) that could have influenced PAR through 26 possible differentially expressed receptors (Fig. 8). When assessing the role of immune cells in the cross-talk between PAR and MFP, 4 PAR (*CXCL5*, *IL1B*, *CXCL10*, and *CXCL9*) (Fig. 9) and 3 MFP (*CSF1*, *NGF*, and *TGFB1*) (Fig. 10) differentially expressed protein coding genes were involved in the tissues cross-talk.

## Discussion

Over the past decades, calf nutrition research has focused on developing strategies that facilitate early weaning, delivering a smooth transition from liquid to solid feed. The main focus has been on increasing calf starter intake by reducing milk supply (~ 10% BW), hence facilitating weaning at 7–8 wk., and possibly improving early rumen development and function [33]. However, recent work revealed poor weight gains, higher risk of disease, abnormal behavior, and a reduction in calf welfare when restricted-feeding strategies are used [33]. In contrast, providing greater quantities of milk or MR improves growth and feed efficiency [33]. Furthermore, conventional MR is composed of 20–22% of crude protein, but calves respond better to increased milk allowance when MR contains higher protein and lower fat (up to 30%, with 15–20% fat) [33]. This feeding strategy has been recently described as “accelerated early nutrition”, “accelerated growth”, “enhanced nutrition”, “intensified nutrition”, or “biologically appropriate growth” [34]. The current manuscript compares the effect of a restricted-style management strategy, with an enhanced one on calf parenchyma and mammary fat pad transcriptome.

### The mammary parenchyma

#### The activity of mammary parenchyma

We previously showed that, compared with R, EH calves had greater mammary PAR mass (7.3 fold greater) without changes in PAR composition (e.g. protein or fat concentration) [23]. Furthermore, incorporation of bromo-2′-deoxyuridine indicated greater (~ 60% higher) mammary epithelial cell proliferation in EH than R calves, particularly at the terminal end of the developing ducts (terminally ductal units) [35]. There results were both mirrored at a transcriptome level. The IPA-predicted functions highlighted greater proliferation and differentiation activity of PAR cells (e.g. quantity, proliferation, and differentiation of cells; Fig. 4). The DIA analysis further confirmed the IPA prediction, as greater upregulation of pathways responsible for cell growth, DNA replication and repair, and expression and translation of genetic information was observed. Furthermore, up-regulation of pathways such as ‘cell adhesion molecules’, ‘ECM-receptor interaction’, ‘focal adhesion’, ‘gap junction’, and ‘adherens junction’ illustrate the formation of a cohesive tissue with cell communication and interaction mechanisms in place. Cell-to-cell signaling by contact is a well-known stimulus of cellular proliferation via a PI3K-dependent intracellular signal [36], a cascade that both DIA and IPA analysis predict to be greatly activated in EH calves.

The dissection of the harvested samples focused on the isolation of the mammary PAR. However, the complex nature of the mammary gland tissue will yield a sample mostly composed of PAR cells, together with a heterogeneous ensemble of other tissue types (e.g., nervous, connective). This heterogeneity was detected by the bioinformatics analysis, supporting the notion that the mammary gland is growing as an organ, rather than just due to the proliferation of PAR cells. In fact, both IPA (Fig. 4) and DIA (e.g. ‘axon guidance’ pathway, and all nervous system subcategory pathways) analysis highlighted the growth and development of the nervous system. Furthermore, the up-regulation of pathways such as ‘renin-angiotensin system’ and ‘vascular smooth muscle contraction’ indicate an increased development of the circulatory system, all supported by the activation of the ‘VEGF (vascular endothelial growth factor) signaling pathway’, master regulator of vascular development and of blood and lymphatic vessel function [37].

#### Nutrient supply and metabolism

A constant and abundant supply of nutrients to allow for the rapid growth of mammary PAR was ensured by the up-regulation in EH calves of the ‘ABC transporters’ (ATP binding cassettes transporters), a large family of macro- and micro-nutrients active transporters [38]. At the same time, the up-regulation of ‘Endocytosis’ and ‘Lysozome’ pathway allow for the use of extracellular matrix protein (an important alternative source of nutrients) when accessible to cells [39]. Furthermore, the up-regulation of the ‘mTOR signaling pathway’ and almost all amino acid metabolism pathways would have helped sustain the protein synthesis machinery of the proliferating tissue, which took advantage of the increase intake of protein provided by the EH MR. Further discussion on amino acid metabolism in the PAR can be found in Additional file 5.

Besides amino acids and proteins as building blocks, proliferating cells require a substantial amount of energy to support neo-synthesis of proteins, and their metabolic activity as a whole [40]. The activation of carbohydrate and lipid metabolism pathways suggests a greater flux of carbon through the citric acid cycle to generate ATP. The increased amount of fatty acid intake via MR likely supplied substrate for beta-oxidation to generate energy, including phosphatidylcholine that was degraded though the alpha-linoleic acid metabolism pathway. Overall, despite the potential increase in the flux through the citric acid cycle, thanks to the supply of acetyl-CoA, the activation of ‘synthesis and degradation of ketone bodies’ suggests a saturation of the cycle’s oxidative capacity. The mammary gland of ruminants is, in fact, capable of ketogenesis even under normal/non-ketogenic conditions [41].

#### Signaling

The tissue growth observed both phenotypically [23] and at the transcription level was induced by many different, but interrelated, signaling pathways, i.e. MAPK, Hedgehog, Wnt, and Phosphatidylinositol signaling pathway. In all tissues including mammary gland, MAPK plays an important role in complex cellular programs such as proliferation, differentiation, development, transformation, and apoptosis [42]. At least three MAPK families have been characterized: extracellular signal-regulated kinase (ERK), Jun kinase (JNK/SAPK) and p38 MAPK [42]. When feeding a higher plane of nutrition, the pattern of DEG in the pathways and the IPA up-stream regulator analysis revealed up-regulation and activation of all three families. Furthermore, the IPA up-stream regulator analysis predicted the activation of other players in these cascades (i.e., pKa, MEK, Akt; Table 3).

The classical Wnt and Hedgehog signaling pathways, both up-regulated in EH calves, have long been known to direct growth and patterning during embryonic development [43], but their activity in the development of the mammary gland was also observed postnatally [44, 45]. The activation of ‘Phosphatidylinositol signaling pathway’ in EH calves, together with the predicted activation of the PI3K complex and Akt group by IPA, underscore the influence of the PI3K/Akt cascade in the regulation of cell proliferation and differentiation. Akt integrates a plethora of extracellular signals to generate diverse outcomes, including proliferation, motility, growth, glucose homeostasis, survival, and cell death [46].

It is likely that many transcription factors (**TF**), or transcription regulators, at the end of these signaling cascades were activated. Despite not having measured the activation of specific TF directly in our experiment, bioinformatics analysis allowed us to predict activation of eight TF, all possible down-stream targets of one or more of the three cascades. Each has a role in cell proliferation and differentiation, and mammary gland development: EGR1 [47], FOXM1 [48], MYC [49], NFKB1 [50], NOTCH1 [51], SMAD2 and SMAD3 [52], and SP1 [53].

The DIA and the IPA up-stream regulator analysis revealed a spectrum of possible hormones, growth factors, and endogenous chemicals that could have acted as systemic or local mediators of responses linked to the EH MR (e.g., insulin and GH-IGF-1 axis, GnRH, androgen, ANG, EGF, FGF2, NRG1, hyaluronic acid, and tretinoin). A complete discussion of their role and involvement in mediating the nutritional effect of an EH MR can be found in Additional file 5.

### Mammary fat pad

Mammary fat pad is essential for development of the secretory epithelium, providing signals that mediate ductal morphogenesis and, probably, alveolar differentiation [54]. As for all other adipose depots of the animal, the adipocytes residing in the MFP are very sensitive to nutritional changes [55]. In the current study, the mass of the MFP increased 5.9-fold when feeding an EH MR [23]. Opposite to PAR, which usually grows via hyperplasia (increasing cell number though proliferation) instead of hypertrophy (increasing cell dimension), the mass of MFP is controlled by a balance of hyperplasia and hypertrophy, due to the presence of proliferating pre-adipocytes and mature adipocytes [56, 57].

The primary stimulus of adipose tissue growth is dietary energy [58]. In the current study the difference in dietary energy supply between the two groups was substantial, as indicated by the 3-fold greater fat intake of EH vs R calves [25]. Judging by the phenotypic differences in the MFP, this stimulated both tissue hyperplasia (e.g., greater tissue DNA), and hypertrophy (e.g. greater lipid accumulation) in EH heifer calves [23]. Despite some discordance, overall, the transcriptome and bioinformatics analysis support both scenarios. The markedly greater ingestion of lipids would have led to an increase in long-chain fatty acids in the blood. The IPA analysis predicted the involvement of fatty acids and LDL (low density lipoproteins) in the observed patterns of DEG. Fatty acids stimulate preadipocyte proliferation through PPAR-δ (up-regulated in EH vs R) [59], while LPL stimulates adipocyte differentiation and maturation [60]. The activation of preadipocyte proliferation in the EH calf MFP was also supported by the activation of the ‘Insulin signaling pathway’ [61] and nucleotide metabolism (purine and pyrimidine). The IPA predicted activation of AGT [62, 63], PPGF BB [64], TNF [63], and F2 and VEGFA [65, 66], all signaling molecules that have been shown to stimulate this process.

Prior to actively accumulating lipid, the pre-adipocyte must differentiate and mature into adipocytes. Several molecules, besides LDL, that induce and enhance this process were predicted to be activated in EH calves: Pkc(s) [67], Creb (CREB1 in particular in our results) [68, 69], GH1 [70], FGF2 [71], PDGF BB [64], and PPARG [72]. Furthermore, the TF SMAD7, which blocks premature differentiation [73], was predicted to be inhibited in response to the EH MR, and Wnt and the ‘Wnt signaling pathway’, a cascade known to inhibit commitment to the adipogenic lineage and adipogenesis [74] and to be downregulated by PPARγ [75], were predicted to be down-regulated.

Once the adipocytes have matured, their metabolism shifts to that of typical adipose tissue, accumulating lipids in their droplets and releasing them when needed [56]. When fed an EH MR, the lipid content of the MFP increased ~ 6 fold [23]. This accumulation was mainly due to storage of excess dietary lipid from long-chain fatty acid uptake, or because of intracellular metabolism of the fatty acids such as desaturation as indicated by the activation of ‘Biosynthesis of unsaturated fatty acids’ pathway. Previous data [76] showed no effect of pre-weaning plane of nutrition on blood insulin concentration, but the accumulation of lipids in the current study was probably driven by the activation of the insulin cascade, as suggested by the up-regulation of the ‘Insulin signaling pathway’. Further discussion on the MPF lipid and energy metabolism can be found in Additional file 5.

### The mammary gland as an organ: Tissues interaction and cross-talk

The transcriptome analysis of both PAR and MFP provided great insights on the effect of pre-weaning nutrition in pre-pubertal mammary development. However, despite gaining a holistic perspective on each of its component, the same perspective is lost when considering the mammary gland as a whole organ. In fact, the two tissues are not separate entities, rather collaborative players in the development of the gland. The PAR expansion is of primary interest for the producer in the context of maximizing future milk production. However, the MFP plays a fundamental role in PAR development [77]. In fact, the MFP, together with its various constituents, facilitates many functions of the gland: (i) it houses a vascular and lymphatic system, (ii) it provides a three-dimensional matrix for growth and expansion of the PAR, and (iii) it functions as a local site for hormone action, the provision of lipids, and growth factor synthesis [77].

Regarding growth factors potentially released, the MFP from EH heifers tended to secrete more IGF-1 (1.4x; FDR = 0.11), IGFALS (1.7x; FDR = 0.04), FGF-1 (1.8x, FDR = 0.09), and FGF-2 (1.3x; FDR = 0.09). Of these, only FGF-2, a confirmed mitogen in bovine mammary gland [77], was predicted to be activated by the IPA analysis in both PAR and MFP. Despite IGF-1 not being predicted to be involved in the observed changes in PAR due to EH MR, the up-regulation of its binding proteins IGFALS and IGFBP2, in MFP and PAR, respectively, suggests a possible prolonged effect that might have contributed to the enhanced mammary gland development in EH heifer calves.

#### Immune cell involvement

Recent studies have illustrated the importance of immune cells and their mediators during the various stages of mammary gland development [78].

Information about the involvement of immune cells in mammary development using non-ruminant models has been extensively reviewed elsewhere [78]. In these models, immune cells such as eosinophils, mast cells, macrophages and T-cell occupy unique sites during the various stages of mammary gland development where they contribute to a diversity of effector functions. Bovine specific data [79] suggest these cells cluster closely with the terminal ductal units, clusters of ductules arising from large ducts typical of bovine, in the developing bovine mammary gland. The DIA ‘Immune system’ category, and the IPA canonical pathways (Fig. 6), upstream regulator analysis (Tables 3 and 4), and immune-specific networks (Figs. 7, 8, 9, 10) displayed a substantial involvement of immune-related functions in both tissues. As it is hard to precicely separate the PAR from its surrounding stroma, it is not surprising that expression of immune cell markers for all aformentioned cell-types were detected in both MFP and PAR: *CD4* and *CD32* (general immune cells marker), *CD68* (macrophage), *CD301* (M2-macrophage), *EMR1* (eosinophil), and *FCER1G* (mast cell). Furthermore, *CD11d* (M1-macrophage), *FCER1A* (mast cell), and *CXCR3* (Th1 T cell) were expressed in PAR only. Their expression not only confirms the previously determined involvement of immune cells in dairy cow mammary gland development [79], but results indicated an effect of plane of nutrition on immune cell quantity within each tissue.

According to a model based on non-ruminant species [78], mast cells first localize to the stromal regions surrounding the terminal end buds (TEB), bulb-shaped structures that direct the growth of the ducts throughout the rest of the fat pad. Next, macrophages are recruited and migrate and localize to the neck of TEBs, and lastly, eosinophils are recruited to the head of TEBs. Bovine specific data confirm the recuitment of eosinophils at latter stage of early development, while no clear priority between macrophages and mast cells seemed to emerge [79]. As previously shown by differences in marker expression (*FCER1A* and *FCER1G*), a greater number of mast cells can be hypothesized to reside both in the MFP and around the ducts in the PAR of EH calves. The absence of mast cells was shown to reduce TEBs and number of ducts, and ductal length in mouse, suggesting a fundamental role of this immune cell type in mammary gland development [80]. The mechanism of action is still unclear, but mediators contained in their granules, such as histamine, and serine proteases and their activating enzyme DPPI (dipeptidyl peptidase I), seem to be involved [80]. Bioinformatics analysis indicated that histamine synthesis might have been enhanced in PAR of EH calves, supporting the role of mast cells in the mediation of the greater mammary gland mass in these calves. Furtermore, the mast cell specific triptase beta 2 (a serine protease) was upregulated (1.8x, FDR = 0.05) in PAR of EH heifer calves.

Together with mast cell, macrophages seem to be the primary immune cell type recruited to the bovine mammary gland [79]. *CD68* expression suggested a higher amount in both the MFP and PAR of EH calves, with a tendency for a greater population of anti-inflammatory M2 macrophages in the MFP. Despite being infiltrated in both tissues, the MFP seemed to be primarily responsible for their recruitment. In fact, no changes in the chemokines *CCL2* and *CX3CL1* were detected in PAR, while they were upregulated (*CCL2*, 1.9x, FDR = 0.12; *CXC3CL1*, 1.3x, FDR = 0.05) in MFP of EH heifer calves.

In mice, once recruited by the MFP, macrophages move closer to the neck of the TEBs following the PAR signal through CSF1 (Colony stimulating factor-1), a key player in macrophage proliferation and survival [81]. Here they stimulate ductal elongation through reorganization of surrounding collagen [78]. Indeed, feeding the EH MR increased *CSF1* expression in the MFP (1.4x, FDR = 0.01), but not in the PAR, and this molecule was predicted to be part of the tissue cross-talk network as an MFP signal to the PAR. Despite parenchymal tissue being extraneous to the recruitment of monocytes to the mammary gland, EH feeding increases PAR expression of *CXCL10* (C-X-C Motif Chemokine Ligand 10), a chemokine able to recruit monocytes [82]. As suggested by the generated tissue crosstalk network, this molecule could have affected the MFP, enhancing monocyte recruitment in the surrounding tissue, or could have worked as a local attractant in the PAR to enhance the migration of macrophages from the MFP to the PAR.

Lastly, as previously reported [79], the recruitment of eosinophils to the mammary gland can also be hypothesized in the current animals. In fact, in the MFP, but not in PAR, we were able to detect the expression of *EMR1*, an eosinophil marker. Despite the lack of difference due to plane of nutrition in the expression of this marker, the MFP of EH calves had an upregulation of eotaxin *CCL24* (1.2x, FDR = 0.05), an eosinophil chemoattractant. Furthermore, the IPA predicted the activation of IL5 in the MFP of EH calves. IL5 is a potent eosinophil chemoattractant, which can also increase their activity. In mice lacking either eotaxins or IL5, TEB numbers and ductal branching were impaired [83, 84]. Together with macrophages, eosinophils are a source of TGFβ, a growth factor that not only was predicted to be activated by the IPA in both MFP and PAR, but also was predicted to participate in the crosstalk between MFP and PAR in EH calves. TGFβ might be the primary mediator of eosinophil action on mammary development. This growth factor has an important role in coordinating patterning and growth of the mammary ductal tree, reducing excessive elongation of the single ducts, favoring instead the formation of a complex 3D structure [85]. Besides TGFβ, eosinophils can also release epidermal growth factor (EGF), which supports epithelial cell maintenance during processes of tissue development, repair, and remodeling [78]. EGF was among the predicted activated growth factors involved in the PAR response to an EH MR.

Signs of T-cell infiltration were observed in both PAR and MFP, but differences between nutritional groups were observed only in the PAR, as EH calves had greater expression of *CXCL9*, a T-cell chemoattractant, and *CD3* (subunits ε, 1.3x, FDR = 0.05; and γ, 1.4x, FDR = 0.04), a marker of the T-cell lineage. T helper cell 1 (Th1) cytokines, including IL-2 and interferon-γ (IFNγ), are responsible for directing cell-mediated immunity, including activation of macrophages, while T helper cell 2 (Th2) cytokines such as IL-4, IL-5, IL-6, IL-10, and IL-13 are involved in processes such as matrix deposition and tissue remodeling, all functions needed for mammary gland development [78]. Among these, IL-2, IL-4, IL-5, and INFγ all were predicted to be activated by the IPA, suggesting a role of T-cells in the immune-cell mediated development of the mammary gland induced by an EH MR. Their putative action in the current study was mainly as recruiter and enhancer of the activity of other immune cell types.

## Conclusions

The current bioinformatics analyses of the transcriptome highlight the physiological pathways and signaling cascades that mediate the heifer response to an enhanced MR plane of nutrition in the pre-weaning period. Furthermore, they suggest an increased degree of cross-talk between PAR tissue and the surrounding MFP, with the latter increasing the local release of mammogenic signals and orchestrating the infiltration of immune cells with key roles in the development of the mammary gland.

In light of the current and previous results, enhancing nutrient intake in the pre-weaning period of Holstein heifer calves appears to be a feasible strategy to stimulate mammary development prior to onset of first lactation. Future studies will need to assess lactation performance of calves fed an accelerate milk replacer prior to weaning.

## Additional files

Additional file 1: Complete details of the protocols used for library preparation, primer design and sequences, and qPCR analysis and performance. (DOCX 28 kb)

Additional file 2: Summary of read counts per sample and details of the alignment procedure performed with the STAR 2.5.1b package (DOCX 14 kb)

Additional file 3: Complete result datasets generated by the DIA analysis. The KEGG pathways are sorted by category and subcategory. Blue bars represent the Impact along the transition period, while red and green bars show the Direction of the Impact (red = upregulation, green = downregulation). (XLSX 60 kb)

Additional file 4: Complete list of genes considered in the development of the tissue cross-talk networks between mammary fat pad and parenchyma. (XLSX 37 kb)

Additional file 5: Extended discussion on Parenchymal metabolism and molecular signaling, and Fat Pad lipid and energy metabolism. (DOCX 75 kb)

## Abbreviations

ADF: Acid detergent fiber
AGT: Angiotensinogen
Akt: protein kinase B
ANGPT2: Angiopoietin 2
AR: Androgen receptor
BW: Body weight;
CD11d: Cluster of differentiation 11d
CD301: Cluster of differentiation 301
CD32: Cluster of differentiation 32
CD4: Cluster of differentiation 4
CD68: Cluster of differentiation 68
CP: Crude protein
Creb: cAMP response element binding protein
CSF1: Colony stimulating factor 1
CXCL10: C-X-C motif chemokine ligand 10
CXCL5: C-X-C motif chemokine ligand 5
CXCL9: C-X-C motif chemokine ligand 9
CXCR3: C-X-C motif chemokine receptor 3
DEG: Differentially expressed genes
DIA: Dynamic impact approach
DM: Dry matter
DPPI: Dipeptidyl-peptidase I
EGF: Epidermal growth factor
EGR1: Early growth response 1
EH: Enhanced milk replacer
EMR1: EGF-like module-containing mucin-like hormone receptor-like 1
ERK: Extracellular signal-regulated kinase
F2: coagulant factor II (prothrombin)
FC: Fold change
FCER1A: Fc fragment of IgE receptor Ia
FCER1G: Fc fragment of IgE receptor Ig
FDR: False discovery rate
FGF-1: Fibroblast growth factor 1
FGF2: Fibroblast growth factor 2
FOXM1: Forkhead box M1
FSH: Follicle-stimulating hormone
GH: Growth hormone
GH1: Growth hormone 1
GnRH: Gonadotrophin releasing hormone
IGF-1: Inusulin-like growth factor
IGFALS: Insulin like growth factor binding protein acid labile subunit
IL-10: Interleukin 10
IL-13: Interleukin 13
IL1B: Interleukin 1β
IL-2: Interleukin 2
IL-4: Interleukin 4
IL-5: Interleukin 5
IL-6: Interleukin 6
INFγ: Interferon γ
IPA: Ingenuity pathway analysis
JAK/STAT: Janus kinase/signal transducer and activator of transcription
JNK/SAPK: stress-activated protein kinase/c-Jun NH(2)-terminal kinase
LDL: Low density lioprotein
MAPK: Mitogen-activated protein kinase
MFP: Mammary fat pad
MR: Milk replacer
MTG1: Mitochondrial ribosome associated GTPase 1
MYC: MYC proto-oncogene
NDF: Neutral deternget fiber
NFKB1: Nuclear factor κ B Subunit 1
NGF: Nerve growth factor
NOTCH1: Notch 1
NRG1: Neuregulin 1
PAR: mammary parenchyma
PDGF BB: Platelet-derived growth factor BB
PI3K: Phosphoinositide 3-kinase
Pkc: Protein kinase C
PPAR: Peroxisome proliferator-activated receptor
PPARG: Peroxisome proliferator-activated receptor γ.
PPP1R11: Protein phosphatase 1 regulatory inhibitor subunit 11
R: Restricted milk replacer
RPS15A: Ribosomal protein S15a
SMAD2: SMAD family member 2
SMAD3: SMAD family member 3
SP1: transcription factor Sp1
TDU: Terminal duct unit
TEB: Terminal end bud
TF: Trasncription factor
TGFB1: Transforming growth factor beta 1
TGFβ: Transforming growth factor β
Th1: Type 1 T helper
Th2: Type 2 T helper
TNF: Tumor necrosis factor
VEGFA: Vascular endothelial growth factor A
