# Supplementary material for: Higher plane of nutrition pre-weaning enhances Holstein calf mammary gland development through alterations in the parenchyma and fat pad transcriptome
Source: BMC Genomics. 2018 Dec 11;19:900. doi: 10.1186/s12864-018-5303-8 (PMC6290502; doi:10.1186/s12864-018-5303-8)
Supplement: Supplementary file 1 — Complete details of the protocols used for library preparation, primer design and sequences, and qPCR analysis and performance. (DOCX 28 kb) [file 12864_2018_5303_MOESM1_ESM.docx]

**ADDITIONAL METHODS**

**RNA sequencing**

***Construction of strand-specific.*** Construction of libraries and sequencing on the Illumina HiSeq 4000 were performed at the Roy J. Carver Biotechnology Center at the University of Illinois at Urbana-Champaign. Total RNAs were DNAse-treated with the RNAse-free DNase set from Qiagen and run on a Fragment Analyzer (Advanced Analytical, IA) to evaluate RNA integrity. RNAseq libraries were constructed with the TruSeq Stranded mRNA Sample Prep kit (Illumina, CA). Briefly, polyadenylated messenger RNAs (mRNAs) were enriched from 1µg of high quality DNA-free total RNA with oligodT beads. The mRNAs were chemically fragmented, annealed with a random hexamer and converted to double stranded cDNAs, which were subsequently blunt-ended, 3’-end A-tailed and ligated to unique indexes adaptors. The adaptor-ligated double-stranded cDNAs were amplified by PCR for 8 cycles with the Kapa HiFi polymerase (Kapa Biosystems, MA) to reduce the likeliness of multiple identical reads due to preferential amplification. The final libraries were quantitated with Qubit (ThermoFisher, MA) and the average library fragment length was determined on a Fragment Analyzer. The libraries were diluted to 10nM and further quantitated by qPCR on a CFX Connect Real-Time qPCR system (Biorad, Hercules, CA) for accurate pooling of the barcoded libraries and maximization of number of clusters in the flowcell.

***Sequencing of libraries in the HiSeq 4000.*** The pooled barcoded libraries were loaded on 4 lanes of an 8-lane flowcell for cluster formation and sequenced on an Illumina HiSeq 4000. The libraries were sequenced from one end of the cDNA fragments for a total of 100bp. The fastq read files were generated and demultiplexed with the bcl2fastq v2.20 Conversion Software (Illumina, San Diego, CA). The quality of the resulting fastq files was evaluated with the FastQC software, which generates reports with the quality scores, base composition, k-mer, GC and N contents, sequence duplication levels and overrepresented sequences.

**Validation of Sequencing results**

***cDNA synthesis.*** A portion of the RNA was diluted to 100 ng/μL with DNase/RNase-free water for cDNA synthesis through RT-PCR. Per each sample, 4 μL of diluted RNA were mixed with 40 μL of a master mixed composed of 5 μL of Random Primers (3 μg/μL; cat#11034731001, Roche) and 45 μL of DNase/RNase-free water. The mixture was incubated at 65°C for 5 min and kept on ice for 3 min. A total of 36 μL of a second master mix, composed of 20 μL of 5X First-Strand Buffer (cat#EP0441, Thermo Scientific), 5 μL of Oligo dT18 (Custom DNA Oligo Tubes, Integrated DNA Technologies), 10 μL of 10mM dNTP mix (cat#18427-088, Invitrogen), 1.25 μL of Reverse Transcriptase (200 U/μL as final concentration, cat#EP0441, Thermo Scientific), 0.625 μL of Rnase inhibitor (200 U/μL as final concentration, cat#EO0381, Thermo Scientific), and 8.125 μL of DNase/RNase-free water, was added and samples were incubated at 25°C for 5 min, 42°C for 60 min, and 70°C for 5 min. Both reactions were performed in an Eppendorf Mastercycler Gradient. The cDNA was then diluted 1:4 with DNase/RNase-free water, prior to Quantitative Polymerase Chain Reaction (qPCR) Analysis. Prior to dilution cDNA aliquots from each sample were combined to create a pool used for primer testing and to prepare the relative standard curve used during qPCR analysis.

***Primer design and testing.*** A total of 8 genes per tissue (4 up-regulated, and 4 down-regulated) were chosen among the most differentially expressed genes (e.g., those with greatest fold change). Primer were design using the NCBI Primer-BLAST tool ( <https://www.ncbi.nlm.nih.gov/tools/primer-blast/index.cgi>) inputting specific mRNA accession, setting minimum and maximum product length to 100 and 150 bases, respectively, limiting the primer pair specificity to the Bos Taurus organism (taxid: 9913), and forcing an exon junction span. Other parameters were maintained as default. The design primer pairs were then subjected to testing. A mix of 8 μL of diluted cDNA pool (1:4, with DNase/RNase-free water), 10 μL of SybrGreen (Cat. 95073, Quanta Bio), and 1 μL each of the forward and reverse primer (100 uM) were incubated in an Eppendorf Mastercycler Gradient at 50°C for 2 min, 95°C for 10 min, and 40 cycles at 95°C for 15 sec followed by 1 min at 60°C. The lid was set at 100°C for the entire run. At the end of the run an aliquot of 5 μL was mixed with 2 μL of loading buffer (Cat. 8546G, Ambion) and run on a 2% agarose gel, with 0.026‰ of SybrSafe (Cat. S33102, Invitrogen). After the presence of a single band of the right dimension (depending on primer pair design) was confirmed using a BioRad Chemidoc MP imager, the PCR product was purified from the remaining 15 μL using the Qiaquick® PCR Purification kit (Cat. 28104, Qiagen), following manufacturer instruction. The purified product was then sequence via Sanger sequencer at the Roy J. Carver Biotechnology Center at the University of Illinois at Urbana-Champaign. The obtained sequenced was then blasted against the bovine genome to verify primer specificity. Primer pair sequences, product size, and product sequence are reported in Table 1 and 2.

***qPCR analysis.*** Quantitative PCR was performed using 4 μL diluted cDNA combined with 6 μL of a mixture composed of 5 μL SYBR Green master mix (Cat. 95073, Quanta Bio), 0.4 μL each of 10 μM forward and reverse primers, and 0.2 μL DNase/RNase free water in a MicroAmp™ Optical 384-Well Reaction Plate (Applied Biosystems). Each sample was run in triplicate and a 7 points relative standard curve plus the non-template control were used. Standard curve was prepared from the cDNA pool with a 1:2 dilution in DNase/RNase free water to obtain the first point, and a 1:5 serial dilution to generate standard point 2 through 7. A relative quantity of 100 was assigned to the first standard point, with the following point quantity adjusted according to the used dilution. The reactions were performed in an QuantStudio™ 7 Flex System instrument using the following conditions: 2 min at 50 °C, 10 min at 95 °C, 40 cycles of 15 s at 95 °C (denaturation), and 1 min at 60 °C (annealing + extension). The presence of a single PCR product was verified by the dissociation protocol using incremental temperatures to 95 °C for 15 s plus 65 °C for 15 s. Data were calculated with the QuantStudio™ Real Time PCR Software interpolating the samples Ct with the relative standard curve, so to generate a relative mRNA abundance. The final data were normalized (e.g. samples relative mRNA abundance ÷ normalization factor) using the geometric mean of three internal control genes (ICG): *MTG1*, *PPP1R11*, and *RPS15A*.These ICG primer information are reported elsewhere [1] qPCR performances are reported in Table 3.

**REFERENCES**

1. Piantoni P, Bionaz M, Graugnard DE, Daniels KM, Akers RM, Loor JJ: **Gene expression ratio stability evaluation in prepubertal bovine mammary tissue from calves fed different milk replacers reveals novel internal controls for quantitative polymerase chain reaction.** *The Journal of Nutrition* 2008, **138**(6):1158-1164.

**Table 1.** Forward and Reverse Primer sequence use to perform qPCR analysis on the target genes chosen for validation of RNA sequencing results.

| **Gene Symbol** | **Entrez ID** | **Sequence** | **Product Length** |
| --- | --- | --- | --- |
| *Mammary Fat Pad* | | | |
| *CAV3* | 615310 | F: GCCCAGATCGTCAAGGACAT | 102 |
|  |  | R: CACGTCTTCAAAATCCACCTTCA |  |
| *ELN* | 280781 | F: CTGCCAGGTGGCTATGGACT | 103 |
|  |  | R: CCGTTGGATACCCAGCCTTG |  |
| *FCGR2A* | 782652 | F: AGTCACAGAGGCCAGTACCA | 102 |
|  |  | R: TGCTGGACCTTCATTCCCAT |  |
| *LOC781726* | 781726 | F: TCCAGTTGGAATATCCCTTTGTGA | 173 |
|  |  | R: ACAAAACTTCTGCCCCCTGAA |  |
| *PLIN4* | 107131384 | F: TGCTGCCTTCAGACAAGATGA | 127 |
|  |  | R: CCCTGGACCACACCTTTAGC |  |
| *PLPPR4* | 513388 | F: GCTTTTATTTCGTGGAGTTGCC | [126](https://www.ncbi.nlm.nih.gov/nucleotide/1387262719?report=gbwithparts) |
|  |  | R: GCATGCTAAGACTCCGGTCA |  |
| *RPRML* | 616711 | F: GCTCCTTGGGGCGATTTTTC | 118 |
|  |  | R: GTGTCCCAAGGTTGGAAGGG |  |
| *WDR66* | 536738 | F: CCCATCGGAAGGGGAGATTG | 137 |
|  |  | R: TACCGAAGGGTGGCCTATGA |  |
| *Mammary Parenchyma* | | | |
| *ABCC11* | 101909228 | F: TCTCCTACCTCACGCTGTCA | 129 |
|  |  | R: GACGAAGCCTTTTGGCGTTT |  |
| *ADAM12* | 407229 | F: AGGAAGAACTGCCATTGCGA | 108 |
|  |  | R: ACCCTGGTTATCTGCTTGCC |  |
| *ASPHD1* | 614735 | F: TGGCTCATAATGGCTCTCCC | 123 |
|  |  | R: TTCAAGGGTCTGGTGCGAAG |  |
| *LOC100335879* | 100335879 | F: GCGTTCTCTTCCCAACTGGA | 112 |
|  |  | R: AGCTTGCACTCTTCGCTCTT |  |
| *MPZ* | 539462 | F: GGACTATGGTGACAACGGCA | 114 |
|  |  | R: CCGTACCTCGTAGGCACTTT |  |
| *PLN* | 100125240 | F: CACAACAGCCAAGGCTACCA | 133 |
|  |  | R: GCAGAGCGAGTGAGGTACTG |  |
| *SCN7A* | 507516 | F: TCAGATGCTAGTCAGTGTCCTG | 140 |
|  |  | R: GCTTCAGGGTAATCCTGCGT |  |
| *SEZ6L* | 520525 | F: ATCCAGTACACCTGCAACCC | 124 |
|  |  | R: GGGACTCCTCCGAAACACAG |  |

**Table 2.** Sequencing results of the product generated by each primer pair

| **Gene Symbol** | **Sequence** | **Accession #** |
| --- | --- | --- |
| *Mammary Fat Pad* | | |
| *CAV3* | TAGAACATCAACGAGGACATAGTGAAGGTGGATTTTGAAGACGCTGA | NM_001046558.1 |
| *ELN* | GGCCTGGAGGAGTGGCTGGTGCCGCGGGCAAGGCTGGGTATCCAACGGA | NM_175772.2 |
| *FCGR2A* | CTCATCACAGCCAGTGAACATCACTGTCCAAGATGGGAATGAAGGTCCAGCAATT | NM_001109806.1 |
| *LOC781726* | CATTCTGGGGAGGCCATAGGAAGCTCTTTGAGAGGAGCTTTAGGAGGACTGGATTTT  GTGAGTATCCAGAATCATTCCTGTGTACCTGCACCAGACAGCGGCTTCAGGGGGCA  GAAGTTTTGTA | XM_010802825.3 |
| *PLIN4* | GCCAGGTACCAAGGATGCTCTCTCCACAGGAATGGCTAGTATCGTGGATACTGCTAAA  GGTGTGGTCCAGGGATCATCTTGTCTGAAGGCAGCAA | XM_024995015.1 |
| *PLPPR4* | TATTTCCTTGACTCACGGATGTCTTCAAACCCGTGCACTCTGGATTCAGCTGCTATGAC  CGGAGTCTTAGCATGCA | XM_003585864.5 |
| *RPRML* | TCAGCTTCGCCGACTGAGGGCGGGGCTGCCCTGATCTCCCGACCCCTTCCAACCTTGGG  ACACAA | XM_002696055.5 |
| *WDR66* | TTAGTGAGTATGTGGACACTGGAAGCTCACCGACAAAATCAACTTACCAGATTTCTTCA  AAGTCTACCTCAATCATAGGCCACCCTTCGGTAA | XM_010814029.3 |
| *Mammary Parenchyma* | | |
| *ABCC11* | GGAGAGCTTAGATGAGGACAACATCCCCCAGCTGTCAGTACACGATGCTGCAGACAAAA  ACGCCAAAAGGCTTCGTCA | XM_024978937.1 |
| *ADAM12* | TCGGAGGGAGCACGGACAGCGGCCCCGTTCCGGCAAGCAGATTAACCTAGGGTA | NM_001001156.1 |
| *ASPHD1* | ACTCTGGCATCCCAACGTGGCTGGGGCCGAACGCCAGGCCCTCGACTCTTGTCATTCGCA  CCAGACCCTTGAAA | NM_001081741.1 |
| *LOC100335879* | TGGCAGAGAGAGTGCTCCGAGGGCTCCCTGGACCTGCAAAGCTCCCGAAGAGCGAAGAG  TGGCAAGC | XR_237661.4 |
| *MPZ* | GGCAAGACCTCTCAGGTCACGCTCTATGTCTTTGAAAAAGTGCCTACGAGGTACGGA | NM_001079507.2 |
| *PLN* | CTTTTTATCTTTCTCTTGAGCATTTGAAACTCCGGGCTCCCTGCCTTCCTGGTACCATGGAT  AAAGTCCAGTACCTCACTCGCTCTGC | NM_001103319.1 |
| *SCN7A* | TCCTGATGATGGCTACACAAATTTTGACAGCTTTGGCTGGGCCTTATTAGCTCTATTTCGGT  TAATGACGCAGGATTACCCTGAAGCA | XM_010802033.3 |
| *SEZ6L* | CTACAGCCGCGAGACCGGCACCCCCATCTGGGACCTCTCGCCTGCCCCACTGTG  TTTCGGAGGAGTCCCA | XM_024977512.1 |

**Table 3.** qPCR performance data for each analyzed target

| **Target** | **Median Ct^1^** | **Median ∆Ct^2^** | **Slope^3^** | **(R^2^)^4^** | **Efficiency**^5^ | |
| --- | --- | --- | --- | --- | --- | --- |
| *Mammary Fat Pad* | | | | | |  |
| *CAV3* | 28.77 | 6.90 | -3.226 | 0.978 | 2.042 | |
| *ELN* | 18.85 | -2.64 | -3.433 | 0.998 | 1.956 | |
| *FCGR2A* | 27.73 | 6.29 | -3.148 | 0.949 | 2.078 | |
| *LOC781726* | 35.54 | 13.96 | -4.436 | 1.000 | 1.680 | |
| *PLIN4* | 20.29 | -1.12 | -3.318 | 0.997 | 2.001 | |
| *PLPPR4* | 27.50 | 5.65 | -3.363 | 0.979 | 1.983 | |
| *RPRML* | 30.94 | 8.80 | -3.169 | 0.978 | 2.068 | |
| *WDR66* | 31.31 | 9.89 | -2.843 | 0.996 | 2.247 | |
| *Mammary Parenchyma* | | | | | |  |
| *ABCC11* | 25.79 | 3.79 | -3.312 | 0.989 | 2.004 | |
| *ADAM12* | 20.91 | -0.34 | -3.270 | 0.997 | 2.022 | |
| *ASPHD1* | 27.43 | 6.06 | -3.414 | 0.955 | 1.963 | |
| *LOC100335879* | 28.58 | 7.40 | -3.098 | 0.993 | 2.103 | |
| *MPZ* | 23.86 | 1.88 | -3.257 | 0.995 | 2.028 | |
| *PLN* | 25.49 | 4.12 | -3.241 | 0.985 | 2.035 | |
| *SCN7A* | 28.03 | 6.68 | -3.337 | 0.991 | 1.994 | |
| *SEZ6L* | 29.13 | 8.07 | -3.479 | 0.996 | 1.938 | |
| ^1^ The median is calculated considering all samples.  ^2^ The ∆Ct is calculated as [Ct gene – geometrical mean of Ct internal controls] for each sample. The median is then calculated considering all samples.  ^3^ Slope of the standard curve.  ^4^ R^2^ stands for the coefficient of determination of the standard curve.  ^5^ Efficiency is calculated as [10^(-1 / Slope)^]. | | | | | |  |
