# Supplementary material for: Higher plane of nutrition pre-weaning enhances Holstein calf mammary gland development through alterations in the parenchyma and fat pad transcriptome
Source: BMC Genomics. 2018 Dec 11;19:900. doi: 10.1186/s12864-018-5303-8 (PMC6290502; doi:10.1186/s12864-018-5303-8)
Supplement: Supplementary file 2 — Summary of read counts per sample and details of the alignment procedure performed with the STAR 2.5.1b package (DOCX 14 kb) [file 12864_2018_5303_MOESM2_ESM.docx]

|  |  |  |  | **% of reads unmapped** | | |
| --- | --- | --- | --- | --- | --- | --- |
| **Sample** | **Total reads** | **Uniquely mapped reads** | **Reads mapped to multiple loci** | **too many mismatches** | **too short** | **other** |
| S2_btMFP_EH | 22,883,157 | 21,254,606 (92.88%) | 911,917 (3.99%) | 0.00% | 2.86% | 0.09% |
| S3_btMFP_R | 26,108,899 | 23,427,035 (89.73%) | 1,492,349 (5.72%) | 0.00% | 4.20% | 0.09% |
| S4_btMFP_EH | 26,880,034 | 25,105,265 (93.4%) | 1,067,754 (3.97%) | 0.00% | 2.34% | 0.10% |
| S5_btMFP_R | 30,504,713 | 27,587,527 (90.44%) | 1,639,808 (5.38%) | 0.00% | 3.85% | 0.09% |
| S6_btMFP_R | 29,435,423 | 27,176,687 (92.33%) | 1,385,835 (4.71%) | 0.00% | 2.65% | 0.10% |
| S7_btMFP_R | 31,553,237 | 28,981,374 (91.85%) | 1,554,102 (4.93%) | 0.00% | 2.93% | 0.08% |
| S8_btMFP_EH | 34,521,650 | 32,098,685 (92.98%) | 1,403,212 (4.06%) | 0.00% | 2.67% | 0.10% |
| S9_btMFP_R | 24,155,833 | 22,063,169 (91.34%) | 1,155,805 (4.78%) | 0.00% | 3.50% | 0.12% |
| S10_btMFP_R | 25,090,266 | 23,162,749 (92.32%) | 1,258,207 (5.01%) | 0.00% | 2.37% | 0.08% |
| S1_btMFP_EH | 25,015,015 | 23,336,533 (93.29%) | 974,628 (3.9%) | 0.00% | 2.51% | 0.11% |
| S11_btMFP_EH | 31,109,720 | 28,926,559 (92.98%) | 1,265,372 (4.07%) | 0.00% | 2.68% | 0.09% |
| S12_btPAR_EH | 27,322,256 | 25,428,776 (93.07%) | 1,291,814 (4.73%) | 0.00% | 1.80% | 0.15% |
| S13_btPAR_R | 24,428,569 | 22,205,100 (90.9%) | 1,196,783 (4.9%) | 0.00% | 3.86% | 0.10% |
| S14_btPAR_EH | 27,086,904 | 25,250,667 (93.22%) | 1,227,878 (4.53%) | 0.00% | 1.91% | 0.12% |
| S15_btPAR_R | 32,517,072 | 29,863,750 (91.84%) | 1,609,111 (4.95%) | 0.00% | 2.87% | 0.11% |
| S16_btPAR_R | 30,160,662 | 28,021,992 (92.91%) | 1,360,524 (4.51%) | 0.00% | 2.27% | 0.10% |
| S17_btPAR_R | 30,290,995 | 27,758,654 (91.64%) | 1,476,092 (4.87%) | 0.00% | 3.16% | 0.09% |
| S18_btPAR_EH | 26,355,753 | 24,657,066 (93.55%) | 1,182,702 (4.49%) | 0.00% | 1.61% | 0.13% |
| S19_btPAR_R | 20,953,947 | 19,281,265 (92.02%) | 975,302 (4.65%) | 0.00% | 2.98% | 0.12% |
| S20_btPAR_R | 21,507,051 | 19,963,664 (92.82%) | 983,049 (4.57%) | 0.00% | 2.24% | 0.13% |
| S21_btPAR_EH | 24,444,837 | 22,633,584 (92.59%) | 1,122,966 (4.59%) | 0.00% | 2.49% | 0.10% |
| S22_btPAR_EH | 27,787,745 | 26,099,687 (93.93%) | 1,258,016 (4.53%) | 0.00% | 1.14% | 0.17% |
