# Supplementary material for: Higher plane of nutrition pre-weaning enhances Holstein calf mammary gland development through alterations in the parenchyma and fat pad transcriptome
Source: BMC Genomics. 2018 Dec 11;19:900. doi: 10.1186/s12864-018-5303-8 (PMC6290502; doi:10.1186/s12864-018-5303-8)
Supplement: Supplementary file 5 — Extended discussion on Parenchymal metabolism and molecular signaling, and Fat Pad lipid and energy metabolism. (DOCX 75 kb) [file 12864_2018_5303_MOESM5_ESM.docx]

**Additional File 4**

**Extended discussion on Parenchymal metabolism and molecular signaling, and Fat Pad lipid metabolism.**

**Amino acid metabolism in the parenchyma.**

Among the amino acid metabolism pathways, 4 were among the top-20 most impacted pathways: phenylalanine, histidine, tryptophan, and tyrosine metabolism. Besides the classic metabolism of tyrosine to the neurotransmitters DOPA and dopamine [1], the up-regulated DEG in the closely-related phenylalanine and tyrosine metabolism pathways suggest their use for energy via oxidation through the citrate cycle. Exploring the DEG patterns of the pathways indicated that the activation of histidine metabolism in EH heifers led to the synthesis and catabolism of histamine, which probably increased blood vessel permeability favoring the movement of nutrients to the proliferating cells. Furthermore, it certainly allowed for easy extravasation of immune cells, an important step in mammary development discussed later. The DEG pattern in the metabolism of tryptophan instead likely led to higher synthesis of serotonin in the mammary gland of EH heifers. Traditionally, serotonin is highly synthetized in intestinal cells and the central nervous system, but recent work in mice demonstrated the mammary epithelium can synthetize this hormone [2]. Its role in bovine mammary gland has recently been studied in adult lactating animals, with a role in involution of the secretory tissue [2, 3]. No data are available, to our knowledge, about its effect during development of the pre-pubertal cow, but work in mice emphasizes its role in cell homeostasis as a tight-junction regulator [4, 5], which could be functionally important during development.

As indicated by the up-regulation of its metabolism, methionine utilization seems to respond to EH MR. Furthermore, the up-regulation of ‘Folate biosynthesis’ and ‘One carbon pool by folate’ indicates an increase in synthesis of tetrahydrofolate (THF) and its subsequent methylation to 5-methyl-THF, which is used by cells to regenerate methionine from homocysteine [6]. Methionine also could be used to synthetize the antioxidants glutathione or taurine [6]. The net flux in the metabolism of the first end product, and the positive flux of the second, indicate potentially greater synthesis of taurine. However, the down-regulation of glutathione peroxidase 1 (*GPX1*) (a key enzyme in the response to oxidative stress) and the up-regulation of glutathione S-transferase M1 (*GSTM1*) (an initiator of glutathione degradation) suggest that the synthesis of taurine was not driven to respond to an increase in concentration of free radicals.

Serving as the precursor of the two major cell antioxidants does not seem the main function of methionine affected by the EH MR. Instead, the increased expression of methionine adenosyltransferase *MAT1A* (traditionally liver specific but also found in mammary tissue) [7] suggests a role of pre-weaning nutrition in increasing PAR DNA methylation. In fact, MAT1A catalyzes the synthesis of S-adenosylmethionine (SAM), the main donor of methyl groups in the cell [8]. The hypothetical increase in DNA methylation because of an EH MR relates closely to our initial hypothesis and requires further investigation. In fact, recent work in rats underscored that pregnancy might alter the mammary gland epigenetic state and its receptiveness to pregnancy-related hormones, suggesting that the first pregnancy primes the gland to respond to the hormonal changes that announce succeeding pregnancies [9]. As for pregnancy, despite the decrease in methylation reported previously [9], early life nutrition could leave methylation marks on the mammary PAR which could possibly enhance its response to mammogenic stimuli.

**Extra-cellular signals mediating the parenchymal response to an enhance milk replacer.**

Insulin and the GH-IGF-1 (growth hormone; insulin-like growth factor 1) axis have been the major focus of the response to energy and protein intake in neonatal calves [10-13]. This is because of their well-recognized effects on nutritional physiology, body growth, and most importantly, mammary development [14, 15]. When a higher plane of nutrition was fed to pre-weaned calves, the blood levels of GH and insulin were not affected, while those of IGF-1 increased, with a simultaneous decrease in IGF-1 binding proteins [10]. These hormones were not measured in the current experiment, but the activation of ‘Insulin signaling pathway’, the increased expression of GH receptor (1.4x, FDR = 0.04), and the activation of their intracellular signaling cascades (e.g. MAPK, Akt/Pi3K, JAK/STAT; [14]) strongly suggest their involvement in the enhanced mammary development observed in EH calves. Similarly, the activation of ‘GnRH signaling pathway’ indicates the role of GnRH (gonadotropin releasing hormone) as a potential initiator of the cascades mentioned above. The primary effect of this hormone is exerted in the pituitary gland, but the mammary gland also excretes GnRH and expresses its receptor [16, 17], indicating a potential role for the hormone on mammary biology. Despite the fact that GnRH was suggested to have anti-proliferating effect in extra-pituitary tissues (mainly in cancer models), the fact that its receptors activates all three MAPK cascades during the synthesis of FSH and LH in the pituitary gland [16] seems to suggest a potential role in the growth of mammary PAR, through the MAPK cascade.

Even if the DEG and DIA results support their action, none of the above hormones were predicted as activated by the IPA up-stream analysis. Instead, the action of androgen (testosterone) through the androgen receptor (**AR**), was predicted as possible effector of the response to an EH MR. This was of particular interest since androgen per se should reduce mammary cell proliferation. However, data from an in-vivo mouse model provided evidence of important roles of androgen for mammary gland development though AR-mediated MAPK and estrogen receptor activation [18]. Furthermore, it is important to remember that androgen can be converted into estrogen by the mammary gland [19].

The DIA indicates the down-regulation of the ‘Steroid hormone biosynthesis’ pathway in response to an EH MR. This was, however, due to a down-regulation of genes involved in minor and branching steps of steroid metabolism. Furthermore, the plane of nutrition did not affect the expression of the gene encoding the enzyme responsible for the conversion of androgen to estrogen (aromatase). Thus, further research is needed to understand the possible effect of pre-weaning nutrition on circulating sex-hormone conversion in the developing heifer mammary gland.

We previously discussed DIA results of the current analyses suggesting a structural development of the mammary gland as an organ (e.g., angiogenesis, neural development). The IPA results further support this claim. The predicted activation of angiotensinogen (AGN), a liver-secreted precursor of angiotensin, and angiopoietin 2 (ANGPT2), two potent angiogenic agents [20, 21], suggests their involvement in the development and expansion of circulatory system in the mammary gland. Furthermore, AGN was also found to induce proliferation of the mammary gland [20] making it another potential molecule responsible for the increase mass of the tissue observed in EH heifer calves. The predicted activation of arachidonic acid as an effector, due to its ability to enhance angiogenesis [22], further support this scenario.

A central discovery from the IPA analysis is the enhanced formation of a 3D structure of the parenchymal tissue thanks to cellular differentiation and the branching and elongation of the ductal system. In ruminants, ductal elongation is accomplished through the growth, development, and subsequent extension of highly arborescent terminal ductal units (**TDU**) [23]. These consist initially of solid chords of epithelial cells that penetrate the mammary stroma. As the solid chord of epithelial cells extends into the mammary stroma, lateral outgrowths emerge at closely spaced intervals [24]. Previous work [25] revealed how an increase in energy and protein intake resulted in greater development of the mammary parenchyma of 8 wk old heifer calves, with a more mature structure and morphology (e.g. ducts, stromal space) compared with control counterparts.

In the current study, feeding an EH MR caused the activation of the growth factor EGF, FGF2, and NRG1 (together with the strong impact of the ‘ErbB signaling pathway’), and the molecule hyaluronic acid, which are all involved in the control of ductal branching and elongation [26-28]. Tretinoin (or all-trans retinoate), an important modulator of mammary ductal branching [29], was also predicted to be involved in the observed transcriptome changes. The DIA results further revealed an inhibition of ‘Retinol metabolism’, mainly due to the down-regulation of genes involved in tretinoin metabolism, together with up-regulation of genes involved in its synthesis. All together, these results agree with the activation of glycosaminoglycan biosynthesis and inhibition of their degradation. Work with mice demonstrated how these type of glycans contribute to an important aspect of ductal growth and branching morphogenesis in the mammary gland [30].

**Mammary Fat Pad lipid metabolism.**

The ‘Sphingolipid metabolism’ pathway, which leads to the production of ceramide, was also induced in the MFP of EH heifer calves. Since ceramide reduces insulin sensitivity of adipocytes [31, 32], and other tissues in general, its production was probably up-regulated in mature adipocytes to prevent excess lipid accumulation. The same concept can be applied to the observed up-regulation of glycosphingolipids biosynthesis (both globo, and lacto and neolacto series), whose accumulation in adipocytes is known to reduce adipogenesis and insulin sensitivity [33, 34].

As suggested by the up-regulation of ‘Fatty acid degradation’, ‘Citrate cycle’, and ‘Oxidative phosphorylation’ pathway, the MFP of EH heifer calves not only accumulated the excess fatty acid intake, but also seemed to have used part of it for energy production. The up-regulated DEG within the ‘alpha-Linoleic acid metabolism’ pathway points at phosphatidylcholine as an important dietary lipid that was oxidized for energy, as in the PAR cells. However, as opposed to PAR, in MFP phosphatidylcholine was also used to synthesize choline, as indicated by up-regulation of genes involved in ‘Glycerophospholipid metabolism’. The general increase in lipid oxidation probably led to an increase in local production of free radicals, which, if not controlled, could be detrimental for the MFP and the surrounding cells. As a counter mechanism, the catabolism of glutathione (a major antioxidant) was down-regulated (e.g. glutathione metabolism). Since ‘Folate biosynthesis’ was strongly down-regulated, the results suggest that the choline synthesized from dietary phosphatidylcholine was used as a methyl-donor for the re-synthesis of methionine, a glutathione precursor [6]. However, these processes take place namely in liver and kidney, where the necessary enzyme (betaine homocysteine S-methyltransferase, BHMT) is expressed [6]. The RNA sequencing results, in fact, did not detect the presence of *BHMT* mRNA in the MFP. Surprisingly, it was detected in PAR, thus, further studies are needed to understand its function in this tissue, and the possibility of the existence of a complete methionine cycle in the mammary gland.

**REFERENCE**

1. Cooper JR, Bloom FE, Roth RH: **The biochemical basis of neuropharmacology**, 8th edn. Oxford ; New York: Oxford University Press; 2003.

2. Matsuda M, Imaoka T, Vomachka AJ, Gudelsky GA, Hou Z, Mistry M, Bailey JP, Nieport KM, Walther DJ, Bader M *et al*: **Serotonin regulates mammary gland development via an autocrine-paracrine loop**. *Dev Cell* 2004, **6**(2):193-203.

3. Collier RJ, Hemandez LL, Horseman ND: **Serotonin as a homeostatic regulator of lactation**. *Domestic animal endocrinology* 2012, **43**(2):161-170.

4. Stull MA, Pai V, Vomachka AJ, Marshall AM, Jacob GA, Horseman ND: **Mammary gland homeostasis employs serotonergic regulation of epithelial tight junctions**. *Proceedings of the National Academy of Sciences of the United States of America* 2007, **104**(42):16708-16713.

5. Pai VP, Horseman ND: **Biphasic regulation of mammary epithelial resistance by serotonin through activation of multiple pathways**. *J Biol Chem* 2008, **283**(45):30901-30910.

6. Finkelstein JD: **Methionine metabolism in mammals**. *The Journal of nutritional biochemistry* 1990, **1**(5):228-237.

7. Phuong NT, Kim SK, Im JH, Yang JW, Choi MC, Lim SC, Lee KY, Kim YM, Yoon JH, Kang KW: **Induction of methionine adenosyltransferase 2A in tamoxifen-resistant breast cancer cells**. *Oncotarget* 2016, **7**(12):13902-13916.

8. Mato JM, Alvarez L, Ortiz P, Pajares MA: **S-adenosylmethionine synthesis: Molecular mechanisms and clinical implications**. *Pharmacol Therapeut* 1997, **73**(3):265-280.

9. Dos Santos CO, Dolzhenko E, Hodges E, Smith AD, Hannon GJ: **An epigenetic memory of pregnancy in the mouse mammary gland**. *Cell Rep* 2015, **11**(7):1102-1109.

10. Daniels KM, Hill SR, Knowlton KF, James RE, McGilliard ML, Akers RM: **Effects of milk replacer composition on selected blood metabolites and hormones in preweaned Holstein heifers**. *Journal of dairy science* 2008, **91**(7):2628-2640.

11. Smith JM, Van Amburgh ME, Diaz MC, Lucy MC, Bauman DE: **Effect of nutrient intake on the development of the somatotropic axis and its responsiveness to GH in Holstein bull calves**. *J Anim Sci* 2002, **80**(6):1528-1537.

12. Hammon H, Blum JW: **The somatotropic axis in neonatal calves can be modulated by nutrition, growth hormone, and Long-R3-IGF-I**. *Am J Physiol* 1997, **273**(1 Pt 1):E130-138.

13. Breier BH, Gluckman PD, Bass JJ: **Plasma concentrations of insulin-like growth factor-I and insulin in the infant calf: ontogeny and influence of altered nutrition**. *The Journal of endocrinology* 1988, **119**(1):43-50.

14. Hadsell DL, Bonnette SG: **IGF and insulin action in the mammary gland: lessons from transgenic and knockout models**. *Journal of mammary gland biology and neoplasia* 2000, **5**(1):19-30.

15. Sejrsen K, Purup S, Vestergaard M, Weber MS, Knight CH: **Growth hormone and mammary development**. *Domestic animal endocrinology* 1999, **17**(2-3):117-129.

16. Aguilar-Rojas A, Huerta-Reyes M: **Human gonadotropin-releasing hormone receptor-activated cellular functions and signaling pathways in extra-pituitary tissues and cancer cells (Review)**. *Oncol Rep* 2009, **22**(5):981-990.

17. Ikeda M, Taga M, Sakakibara H, Minaguchi H, Vonderhaar BK: **Detection of messenger RNA for gonadotropin-releasing hormone (GnRH) but not for GnRH receptors in mouse mammary glands**. *Biochemical and biophysical research communications* 1995, **207**(2):800-806.

18. Hickey TE, Robinson JL, Carroll JS, Tilley WD: **Minireview: The androgen receptor in breast tissues: growth inhibitor, tumor suppressor, oncogene?** *Mol Endocrinol* 2012, **26**(8):1252-1267.

19. Simpson ER: **Biology of aromatase in the mammary gland**. *Journal of mammary gland biology and neoplasia* 2000, **5**(3):251-258.

20. Escobar E, Rodriguez-Reyna TS, Arrieta O, Sotelo J: **Angiotensin II, cell proliferation and angiogenesis regulator: biologic and therapeutic implications in cancer**. *Curr Vasc Pharmacol* 2004, **2**(4):385-399.

21. Potente M, Gerhardt H, Carmeliet P: **Basic and therapeutic aspects of angiogenesis**. *Cell* 2011, **146**(6):873-887.

22. Pla AF, Grange C, Antoniotti S, Tomatis C, Merlino A, Bussolati B, Munaron L: **Arachidonic acid-induced Ca2+ entry is involved in early steps of tumor angiogenesis**. *Mol Cancer Res* 2008, **6**(4):535-545.

23. Ellis S, Akers RM, Capuco AV, Safayi S: **TRIENNIAL LACTATION SYMPOSIUM: Bovine mammary epithelial cell lineages and parenchymal development**. *Journal of Animal Science* 2012, **90**(5):1666-1673.

24. Capuco AV, Ellis S, Wood DL, Akers RM, Garrett W: **Postnatal mammary ductal growth: three-dimensional imaging of cell proliferation, effects of estrogen treatment, and expression of steroid receptors in prepubertal calves**. *Tissue Cell* 2002, **34**(3):143-154.

25. Brown EG, Vandehaar MJ, Daniels KM, Liesman JS, Chapin LT, Forrest JW, Akers RM, Pearson RE, Nielsen MS: **Effect of increasing energy and protein intake on mammary development in heifer calves**. *Journal of dairy science* 2005, **88**(2):595-603.

26. Hinck L, Silberstein GB: **Key stages in mammary gland development - The mammary end bud as a motile organ**. *Breast Cancer Research* 2005, **7**(6):245-251.

27. Hynes NE, Watson CJ: **Mammary gland growth factors: roles in normal development and in cancer**. *Cold Spring Harb Perspect Biol* 2010, **2**(8):a003186.

28. Hung MC: **On mammary gland growth factors: roles in normal development and in cancer**. *Cold Spring Harb Perspect Biol* 2012, **4**(8):a013532.

29. Wang YA, Shen K, Wang YL, Brooks SC: **Retinoic acid signaling is required for proper morphogenesis of mammary gland**. *Dev Dynam* 2005, **234**(4):892-899.

30. Silberstein GB, Daniel CW: **Glycosaminoglycans in the basal lamina and extracellular matrix of serially aged mouse mammary ducts**. *Mech Ageing Dev* 1984, **24**(2):151-162.

31. Chaurasia B, Kaddai VA, Lancaster GI, Henstridge DC, Sriram S, Galam DL, Gopalan V, Prakash KN, Velan SS, Bulchand S *et al*: **Adipocyte Ceramides Regulate Subcutaneous Adipose Browning, Inflammation, and Metabolism**. *Cell metabolism* 2016, **24**(6):820-834.

32. Chavez JA, Siddique MM, Wang ST, Ching J, Shayman JA, Summers SA: **Ceramides and glucosylceramides are independent antagonists of insulin signaling**. *J Biol Chem* 2014, **289**(2):723-734.

33. Lingwood CA: **Glycosphingolipid functions**. *Cold Spring Harb Perspect Biol* 2011, **3**(7).

34. van Eijk M, Aten J, Bijl N, Ottenhoff R, van Roomen CP, Dubbelhuis PF, Seeman I, Ghauharali-van der Vlugt K, Overkleeft HS, Arbeeny C *et al*: **Reducing glycosphingolipid content in adipose tissue of obese mice restores insulin sensitivity, adipogenesis and reduces inflammation**. *PLoS One* 2009, **4**(3):e4723.
